# Allosteric coupling between a lipid bilayer and a membrane protein

**DOI:** 10.1016/j.bpj.2025.06.033

**Published:** 2025-06-27

**Authors:** Clarisse Fourel, Yanna Gautier, Alexandre Pozza, François Giraud, Elodie Point, Christel Le Bon, Karine Moncoq, Guillaume Stirnemann, Jérôme Hénin, Ewen Lescop, Laurent J. Catoire

**Affiliations:** 1 Institut de Chimie des Substances Naturelles, CNRS, Université Paris-Saclay, Gif-sur-Yvette, France; 2 Laboratoire de Biochimie des Protéines Membranaires, Université Paris Cité, CNRS, Paris, France; 3 Laboratoire de Biochimie Théorique, Université Paris Cité, CNRS, Paris, France; 4 CPCV, Département de Chimie, École Normale Supérieure, PSL University, Sorbonne Université, CNRS, Paris, France

## Abstract

Biological membranes are complex environments whose functions are closely tied to the dynamic interactions between lipids and proteins. Here, we utilize high-pressure NMR of lipid nanodiscs paired with molecular dynamics simulations to elucidate at the atomic scale the allosteric dialog between the lipid bilayer and a model membrane protein, OmpX. We discover that OmpX delays the gelation process by liquefying the annular shell of lipids through hydrophobic and roughness matching processes at the protein surface. Furthermore, modification of the mechanical properties of the lipid bilayer directly impacts the energy landscape of amino acid side chains at the lipid/protein interface but also unexpectedly at the protein core. Our work highlights a thermodynamically coupled but kinetically uncoupled allosteric pathway linking lipid dynamics with the interior of membrane proteins, directly impacting our understanding of membrane function.

## Significance

Integral membrane proteins (IMPs) make up ∼30% of the mammalian proteome and 60% of all drug targets. They are embedded in complex lipid mixtures, and their function is closely tied to membrane properties. Using high-pressure NMR spectroscopy and molecular dynamics simulations, this study reveals how changes in bulk membrane properties allosterically reshape the energy landscape of an IMP, uncovering a long-range dynamic coupling. These findings offer a new perspective on lipid-protein interactions, with implications for understanding mechanotransduction and how membranes adapt to extreme environments, such as deep-sea pressure. This approach also holds promise for advancing drug design by highlighting the role of lipid-protein dynamics in regulating membrane protein behavior.

## Introduction

Cell and organelle membranes are intricate homeostatic regulatory systems. They are mainly composed of a diverse array of lipids, which interact with a wide variety of integral and peripheral proteins in different cell types, subcompartments of membrane organelles, and metabolic states (1). A fundamental area of research focuses on the regulation of integral membrane proteins (IMPs) by the lipid bilayer (2,3). Until now, most atomistic studies of IMP/lipid interactions have focused on high-affinity IMP/lipid contacts thanks to an ever-increasing number of high-resolution 3D structures. At the same time, the allosteric impact of the collective properties of lipid bilayers on the biological functions of membranes has gained increased attention. This was observed for membrane thickness (4,5,6,7), lipid asymmetry (e.g., (8)), lipid packing density and membrane fluidity/viscosity (9,10,11), membrane compressibility (12), membrane curvature and bending (13), mechanical properties (14,15,16), and lipid liquid-liquid phase separation (17,18,19). However, how these bulk membrane properties influence IMP free energy conformational landscape at the atomic scale is still poorly understood. Conversely, as most of biological membranes are patchy and crowded in MPs (20), MPs can in turn impact the physical properties of the lipid bilayer (e.g., (21,22)) or membrane thickness (e.g., (23)). In other words, how does the protein perturb the lipid behavior and respond to pressure (P)/temperature (T) thermodynamic conditions? Progress in this area is currently limited by the difficulty of concomitantly characterizing lipids and proteins with high time and spatial resolutions, all on an atomic scale.

We propose a combination of three main approaches to address these questions. The first involves using soluble nano-objects (nanodiscs), where the protein is embedded within a lipid bilayer. Combining this with cutting-edge IMP isotope labeling and lipids at natural isotope abundance enables solution-state NMR spectroscopy to reveal atomic details on both lipids and IMP. The second key aspect is the use of hydrostatic pressure to control the dynamics of the lipid phase (24,25,26), including fluid-gel phase transitions, to probe potential correlated motion between proteins and lipids (27). Upon pressurization, lipids tend to adopt a more ordered state by adjusting their shape to accommodate the reduced void volume between them. This structural reorganization affects their dynamics, which can be observed using NMR by monitoring the barotropic changes in signal intensities or volumes. The third cornerstone of our approach is the atomic-level interpretation of the experimental measurements using all-atom molecular dynamics (MD) simulations, which have become indispensable tools in a wide range of biophysical applications.

By combining these three essential components, we investigate the complex relationships between one IMP and lipids at both the molecular and atomic levels. We focus on the collective properties of the annular lipid shell surrounding the protein and its long-distance impact on the IMP conformational energy landscape. We provide a clear rationalization at the atomic and molecular levels of how membrane proteins influence the behavior of lipids, their response to pressure, and the spatial extent of this perturbation. In turn, our strategy highlights the reciprocal impact of lipid-phase transitions on membrane protein structure and dynamics. Unequivocally, our study reveals an allosteric coupling between bilayer mechanics and subtle conformational changes in the membrane protein. These physical changes affect not only the amino acid side chains in direct contact with the lipids, but also those facing the protein core, away from the lipids. Our detailed description of the interplay between lipid phase transitions and IMPs at the atomic/molecular scale aims to advance our understanding of the allosteric effects of lipids on membrane protein function.

## Materials and methods

### Production and purification of recombinant OmpX and MSP1D1 proteins

Bacterial expression in *E. coli* (BL21(DE3) strain) and purification of outer membrane protein X (OmpX) and the lipoprotein MSP1D1 were carried out as described previously in (28) and (29), respectively. For solution-state NMR experiments, OmpX was uniformly deuterated and ^15^N-labeled in M9 minimal media in 100% ^2^H_2_O (^2^H > 99%, EURISO-TOP, Saclay, France) solutions supplemented with 2 g/L of u-[^2^H,^12^C]D-glucose (^2^H ≃ 98%, EURISO-TOP) as the source of carbon and 1 g/L of ^15^NH_4_Cl (^15^N ≃ 98%, EURISO-TOP) as the nitrogen source. Specific labeling with ^13^C and protonated methyls at Ala, Val (*γ*2pro*S*), and Ile ($\delta_{1}$-Ile) residues was achieved by following a published protocol (30,31) (TLAM kit from NMRBio, Albenc, France).

### NMR sample preparation

The nanodiscs were formed by adding the lipoprotein to detergent-solubilized 1,2-dimyristoyl-*sn*-glycero-3-phosphocholine (DMPC) or Δ9-cis-PC phospholipids in the absence or presence of OmpX. The formation of nanodiscs was achieved by trapping the detergent with polystyrene beads (Bio-Beads SM-2 Adsorbent Media, Bio-Rad, Marnes-la-Coquette, France) (29,32). The nanodiscs utilized in this study (based on MSP1D1 lipoprotein) have a diameter of approximately 10 nm, which corresponds to a bilayer area of 4400 Å^2^ (29). Such nanodiscs consist of ∼90 molecules of DMPC per leaflet. In the presence of OmpX, which has an ellipsoidal cross-sectional area of ∼600–700 Å^2^ in the transmembrane region based on the NMR structure in DMPC nanodiscs (PDB: 2M06 (32)), each leaflet contains around 80 molecules of DMPC, thus surrounding the protein by 4 lipid shells.

The NMR buffer solution was 25 mM Tris-HCl (pH 7.4), 50 mM NaCl, 2 mM EDTA in 90% H_2_O/10% D_2_O. OmpX-free and OmpX-containing lipid disc concentrations comprised between 300 and ∼500 *μ*M for a total volume of 300 *μ*L in an alumina-toughened zirconia NMR tube (Daedalus Innovations, Aston Township, USA).

### NMR spectroscopy

Solution-state NMR experiments were performed at 15, 25, and 35°C between 1 and 2500 bar. They were conducted on Avance III HD Bruker spectrometers operating at ^1^H 700 and 950 MHz, equipped with TXO and TCI cryoprobes, respectively. 1D ^1^H experiments were collected using the *zgesgp* Topspin pulseprogram. 2D ^1^H,^13^C SOFAST-HMQC (33) spectra were acquired at 950 MHz (with OmpX-containing DMPC or Δ9-cis-PC nanodiscs and with OmpX-free DMPC nanodiscs) and 700 MHz (with OmpX-free Δ9-cis-PC nanodiscs) with a 200 ms recycling delay, 100 ms acquisition time ($t_{2\;\max}$) in the direct dimension, and 13.4 ms ($t_{1\;\max}$) in the indirect dimension (data size = 256($t_{1}$) × 2456($t_{2}$) complex points). The variable flip angle for the PC9 shape pulse was set to 120°. The number of acquisitions per increment was 64, for a total experiment time of 1 h 22 min. All P31 experiments were conducted at 600 MHz with a TBI probe. Data processing and analysis were performed with TopSpin NMR software.

Pressure was adjusted using an Xtreme-60 Syringe Pump apparatus (Daedalus Innovations, Aston Township, USA). A layer of mineral oils was used as a barrier between the sample and the pressurizing medium, as described in (34). Before NMR data collection at high pressure, a rapid pressure jump to 2500 bar was done for each sample outside the spectrometer to ensure the absence of leakage. The pressure ramp was from 1 to 2500 bar, each 100 or 250 bar. A delay of 15 min was imposed after each pressure change and prior to NMR data collection to allow system equilibration. Kinetic experiments confirmed that the lipid system was equilibrated within this time frame and one-dimensional ^1^H spectra collected before and after 2D data collection were identical, indicating that equilibrium was indeed reached. A complete pressure cycle typically corresponds to ∼24 h of data collection.

### Simulated systems

Systems were built using the CHARMM-GUI Membrane Builder tools (35,36,37,38). Membranes were modeled as infinite bilayer systems. All systems were then constructed using the same protocol: a rectangular box was built with the length of *z* based on a water thickness of 22.5 Å and the length of *x* and *y* equal to 60 Å.

### MD simulations

All MD simulations were performed using NAMD 3 version alpha7 (39) with the Charmm36m all-atom force field (40) and the TIP3P water model. Simulations were carried out under periodic boundary conditions based on rectangular boxes containing a hydrated lipid bilayer (in the *xy* plane, i.e., normal to the *z* axis) embedded or not with OmpX.

We used the atom-pair-specific Lennard-Jones parameters for cation-*π* interactions between choline and phenylalanine/tyrosine/tryptophan residues, also known as “WYF parameters” (41). Hydrogen mass repartitioning (42) was applied, allowing for a 4 fs time step, and all bonds involving hydrogen atoms were maintained rigid. The different systems (pure lipid bilayers and bilayers with OmpX) underwent energy minimization, followed by relaxation in the NPT ensemble (i.e., with a constant number of particles N, constant pressure P, and constant temperature T), at 1 bar and 30.15°C. The files provided by CHARMM-GUI Membrane builder were used for equilibration, consisting of 6 cycles of 90 ns each, with planar and dihedral restraints that are progressively removed over the cycles. The systems were then additionally relaxed for 400 ns for the systems with protein and for 10 ns for the pure lipid systems. The simulations were performed in the NPT ensemble and a combination of pressures (1, 250, 500, 750, and 2000 bar) and temperatures (15, 25, and 35°C) were used to explore the DMPC phase diagram. Langevin thermostat and NAMD Nosé-Hoover Langevin piston (43,44) were used to control the temperature and pressure, respectively. Particle mesh Ewald were set with a grid spacing of 1 Å. Nonbonding interactions were limited to 12.0 Å and smoothing functions were applied beyond 10 Å for both electrostatics and van der Waals forces. Pure lipid systems were simulated for 1 μs and lipid bilayers with protein were simulated for 1.5 μs, with three replicas of each system. This gives a total simulation time of 36 μs for DMPC bilayers and 54 μs for OmpX in DMPC. The control lipid was simulated for 1 μs for the pure lipid bilayer for 9 (P, T) conditions and for 1 μs for the system including the protein (2 (P, T) conditions). Configurations were saved every 100 ps for analysis.

### Trajectory analysis

Trajectories were analyzed using VMD (45) version 1.9.4 and Tcl scripts, followed by Python scripts for data postprocessing. Analyses were performed on the last 300 ns of each simulation, taking into account the time needed for the lipid phase transition to occur.

The box volume was normalized to the condition with the largest volume, i.e., at 1 bar and 35°C. The area per lipid was defined as the area of a leaflet (corresponding to the area of the computational box in the *xy* plane) divided by the number of lipids it contains. Dihedral angles of the lipid acyl chains were used to calculate the *gauche* fraction, which corresponds to the number of *gauche* angles (grouping the *gauche* + (0–120°) and *gauche* - (240–360°) angles (46)) divided by the total number of dihedral angles in the system (11 dihedrals for each lipid chain for all lipids). The area per lipid, as well as the volume of the simulation box, and the *gauche* fraction were expressed as a function of time with running averages calculated using a convolution method with a window of 100 ns. The averages presented in this paper were calculated on the data of each replica and the standard deviations were calculated between the replicas.

Moreover, we calculated the atomic radial pair distribution function g(r) and the number integral $\int_{0}^{r}\rho g\left(r\right)r^{2}dr$ between the central glycerol carbon and each water oxygen atom to quantify the hydration of the lipid head. To do this we used the VMD “measure gofr” function on the last 300 ns of the simulation with the following parameters: delta = 0.1, rmax = 15, usepbc = 1, first = 6999, last = −1 and step = 1.

To quantify the spatial organization of the lipids around the protein, we determined the distributions of lipids around the protein at intervals of 0.1 Å over the last 500 ns of each simulation. For lipids, we considered the central glycerol atom and, for the protein, we considered all heavy atoms of residues engaged in a *β*-sheet structure. This resulted in a distribution function at each (P,T) condition, averaged over the replicas, with an example of such distribution shown in Fig. S1. Based on these distributions, we defined first and second lipid layers as corresponding to the [0:5] Å and [5:9.5] Å intervals, respectively. We chose to keep constant boundary values regardless of the (P,T) conditions, whereas the exact distribution of course shifts in position and intensity as pressure and temperature change.

In addition to the metrics defined above, the effect of the protein on the local membrane environment was assessed by measuring the bilayer thickness. The entire simulation trajectory was centered, aligned, and fitted without changing membrane orientation using the *α* carbons of the structured *β*-sheet portions of the protein. Lipids were marked as belonging to the “upper” or “lower” leaflet according to their position, respectively, to the bilayer midplane. The *x*, *y*, and *z* coordinates of each phosphorus atom were then extracted for each frame of the simulation.

For each leaflet, a 100 × 100 grid was constructed based on the *x*, *y* positions of the phosphorus atoms. *z* positions of the phosphorus atoms were extrapolated by linear interpolation to create a plane corresponding to the surface of the upper and lower leaflet. The instantaneous distance between the upper and lower planes was then calculated and considered as the bilayer thickness. When the protein is inserted into the lipid bilayer, the center of mass of the upper (*z* position atoms >0) and lower (*z* position atoms <0) portions of the protein were extracted and patches were created in the planes to mimic the position of the protein in each leaflet. These patches were based on the ellipsoidal cross section of the barrel of the protein in the bilayer (28). To account for the different layers of lipids characterized around the protein, additional ellipsoidal patches were created with dimensions based on the characterized layers, as described above (Fig. S1). The bilayer thickness was measured every 0.1 ns for the last 300 ns of the simulation.

## Results

**Figure 1:**
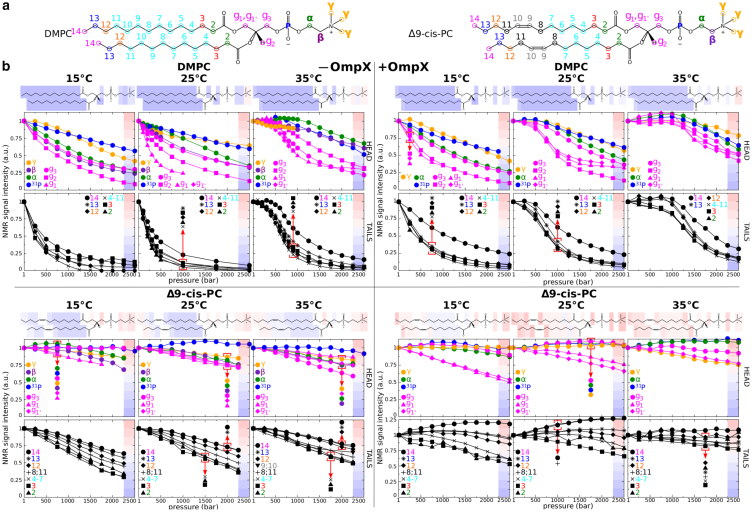
Barotropic behavior of DMPC and Δ9-cis-PC ^1^H NMR signal intensities in nanodiscs in the absence or presence of the protein OmpX. (*a*) Primary chemical structures of DMPC and Δ9-cis-PC with atom nomenclature used in this work. (*b*) Barotropic evolutions of NMR signal intensities at 15, 25, and 35°C observed for DMPC (*upper left* and *right panels*) and Δ9-cis-PC (*lower left* and *right panels*) nanodiscs in the absence (*upper* and *lower left panels*) or presence (*upper* and *lower right panels*) of OmpX. Above each graph is represented a lipid chemical structure overlaid with colored rectangles. The color code corresponds to the signal intensity range per 0.125 of NMR signal intensity observed at the end of the pressure ramp. By convention, all intensities are scaled to 1 at ambient pressure for easy comparison. Temperature/pressure phase properties of DMPC and ^1^H-^13^C NMR assignments of DMPC and Δ9-cis-PC CH_*n*_ in OmpX-devoid nanodiscs are displayed in Figs. S2 and S4, respectively.

All experimental and simulation data presented in this study come from high-resolution NMR spectra of IMP-free or IMP-containing DMPC or 1,2-dimyristoleoyl-*sn*-glycero-3-phosphocholine (Δ9-cis-PC) MSP1D1 nanodiscs. DMPC and its unsaturated analog Δ9-cis-PC have identical structures, except for one additional unsaturation in each acyl chain of Δ9-cis-PC (Fig. 1 *a*). This results in a large decrease in gel/fluid transition temperature for Δ9-cis-PC ($T_{m}$ < 0°C) compared with DMPC ($T_{m}$ ≃ 25°C) at 1 bar (Fig. S2). Simulated data was obtained by explicit all-atom simulations of periodic DMPC or Δ9-cis-PC bilayers, with or without the IMP. For this study, we chose the OmpX from *E. coli* as the model for an IMP, as that protein has been extensively characterized by NMR spectroscopy, including in DMPC nanodiscs of equivalent dimensions at ambient (32) and high pressure (27). OmpX is perdeuterated and contains ^13^CH_3_ specifically incorporated in Ala/Val/Ile residues, allowing us to obtain high-quality 1D ^1^H and 2D ^1^H-^13^C NMR correlation spectra, which simultaneously revealed OmpX and lipid (DMPC or Δ9-cis-PC) behavior (Figs. S3 and S4).

### DMPC and Δ9-cis-PC bilayer phase behavior

We previously demonstrated (27) that ^1^H NMR can detect the fluid-to-gel transition upon pressurization of DMPC in nanodiscs at 40°C. Indeed the fluid-to-gel transition results in major changes in lipid dynamics from a highly mobile phase to a highly rigid phase. Highly mobile and rigid phases are characterized, respectively, by slow and rapid transverse NMR ^1^H spin relaxation, which in turn translates into high- or low-intensity ^1^H peaks. Pressure- or temperature-induced phase transition can therefore be readily detected from the highly sensitive ^1^H 1D spectra, as shown in (Fig. 1 *b*). Here, we expand our exploration of the fluid-gel phase boundary of DMPC in the presence or absence of OmpX using extended temperature (15–35°C) and pressure (1–2500 bar) ranges (Fig. 1 *b*). In the absence of OmpX, a clear phase transition occurs at 35°C (near 600 bar). At 25°C, a slight inflection of the curves is observed around 1 bar, in agreement with the expected transition midpoint of 1 bar at this temperature, whereas at 15°C no such inflection is seen. This is in full agreement with the DMPC phase diagram defined in infinite bilayers (47) (Fig. S2) and previous DSC studies on DMPC nanodiscs (48) that indicate that DMPC bilayers at ambient pressure undergo a transition at 25°C and are in a gel phase at 15°C. The presence of OmpX makes a clear phase transition visible around 1250 bar at 35°C and around 600 bar at 25°C. No transition was observed at 15°C. The use of an extended range of temperature below 40°C was essential to demonstrate the existence of a lipid-phase transition in the presence of OmpX. Indeed, at 40°C, the transition was not detectable within the studied pressure range limited by instrumental constraints, most likely because it is shifted to pressures much larger than 2000 bar (27). This clearly shows that (at least part of) the DMPC lipids surrounding OmpX undergo a cooperative fluid-to-gel transition, but with a phase boundary globally shifted by ∼650 bar when compared with OmpX-devoid nanodiscs. Furthermore, negative controls using Δ9-cis-PC demonstrate no discernible transitions in the presence or absence of OmpX (Fig. 1). Collectively, this strongly suggests that Δ9-cis-PC remains fluid at all tested P/T values, both in the presence or absence of OmpX.

**Figure 2:**
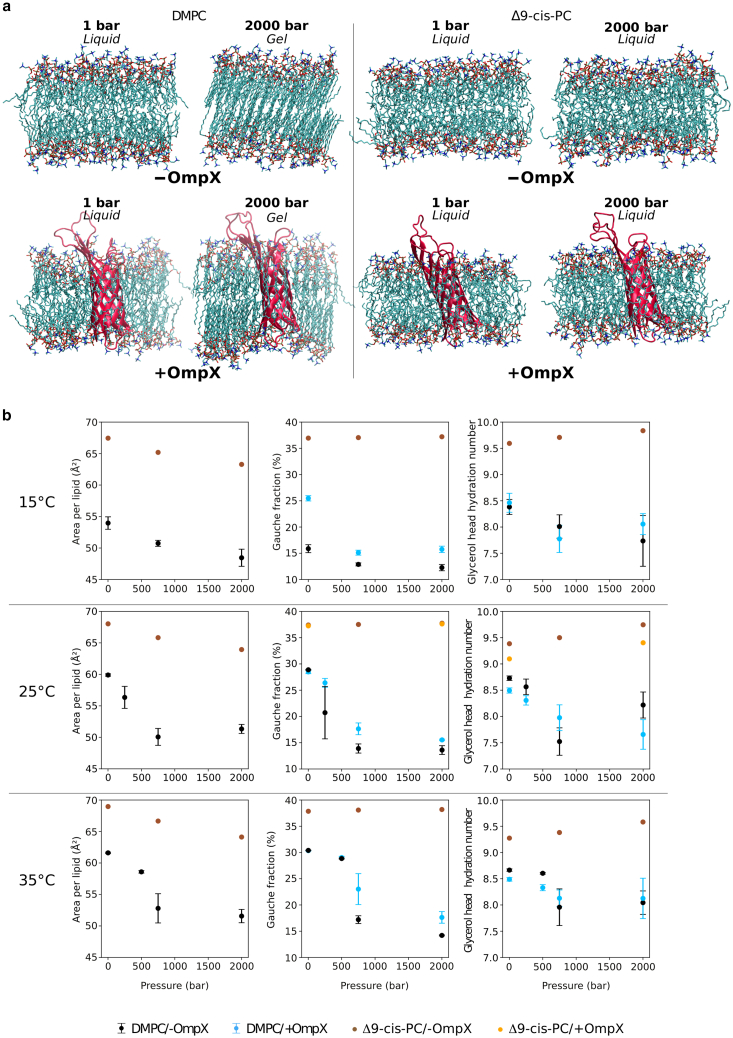
Pressure-induced phase transition of DMPC in the presence and absence of OmpX, characterized by simulations. (*a*) Molecular rendering of simulated systems: pure DMPC (*left*) and Δ9-cis-PC (*right*) bilayers in the absence (*top*) or presence (*bottom*) OmpX (cartoon representation in *red*). In each case, snapshots in thermodynamic conditions corresponding to a liquid state and to a gel phase are shown. (*b*) Area per lipid, *gauche* fraction and glycerol hydration number over the last 300 ns of the simulation as a function of pressure, at a temperature of 15, 25, and 35°C, averaged over three replicas (error bars: standard deviation) for DMPC, and one for Δ9-cis-PC (no error bar).

We gained atomic details on the lipid phase transition and the impact of the insertion of OmpX by carrying out all-atoms MD simulations. These simulations revealed a pressure-induced phase transition consistent with NMR data. We compared low- and high-pressure DMPC systems both with and without an embedded OmpX molecule (Fig. 2). At 25°C, the fluid-to-gel transition in OmpX-devoid DMPC is evident through the sharp decrease in area per lipid (from 60 to 50 Å^2^), *gauche* fraction (from ∼28 to ∼14%), and glycerol head hydration number when pressure increased from 1 to 2000 bar (Fig. 2 *b*), which are all hallmarks of lipid gelation (49). Acyl chain carbon-hydrogen order parameters, another classic reporter of the liquid-gel phase transition, exhibit similar changes along this temperature and pressure scales (Fig. S5). The transition is even sharper at 35°C and occurs between 500 and 750 bar, which perfectly matches the transition observed experimentally by NMR ($P_{m}$ ≃ 600 bar). As a negative control, Δ9-cis-PC does not undergo any transition in simulations.

### Effect of protein on the lipid phase transition: Dynamic plasticity of hydrophobic and roughness matching

**Figure 3:**
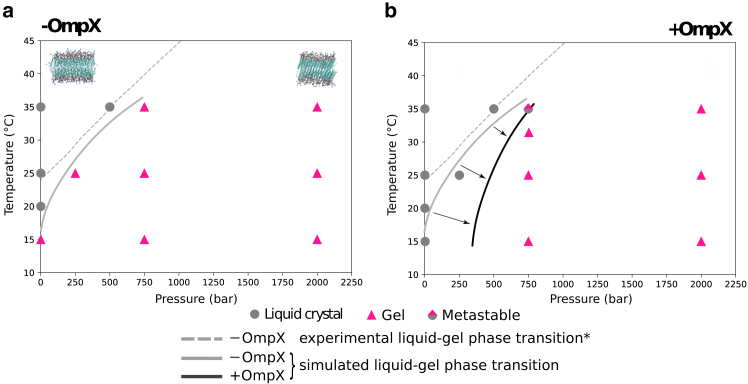
DMPC phase diagram obtained from the MD simulations (*a*) without and (*b*) with OmpX, with symbols corresponding to the liquid, gel, or metastable states of the system in the simulations. ^∗^Corresponds to the experimental DMPC liquid-gel phase transition from Ichimori et al. (47).

MD simulations clearly demonstrate that OmpX affects the lipid dynamics and phase transition of DMPC, shifting the decrease in *gauche* fraction to higher pressures, as observed in the experiments (Fig. 1). The simulated P/T phase diagram in the absence (Fig. 3 *a*) or presence of OmpX (Fig. 3 *b*) is fully consistent with the experimentally determined ones (Fig. 1 *b* and (47)). In the Δ9-cis-PC control system, the *gauche* fraction is almost constant (36%), which is consistent with the total absence of gelation of Δ9-cis-PC around OmpX, as observed by NMR (Fig. 1 *b*).

**Figure 4:**
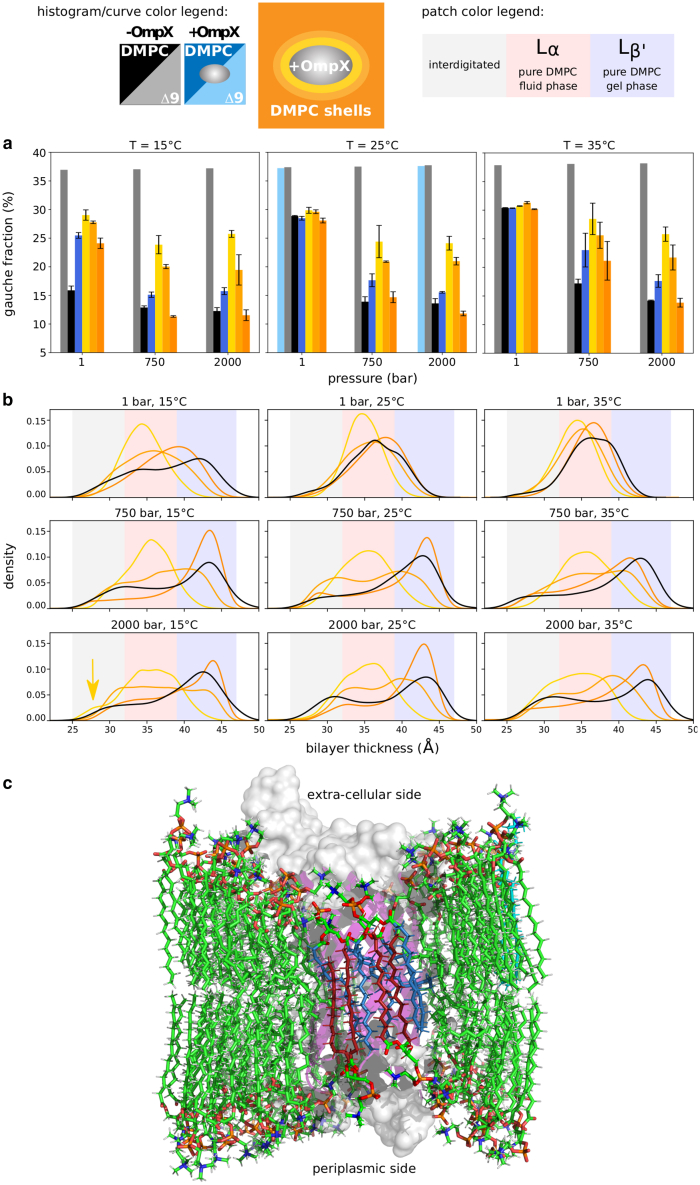
Distance-dependent perturbation of the lipid phase transition by OmpX. (*a*) *gauche* fraction in acyl chains of the pure DMPC (*black*) or Δ9-cis-PC (*gray*) bilayers, in the entire DMPC bilayer with OmpX (*dark blue*) or Δ9-cis-PC with OmpX (*light blue*), and in solvation shells of OmpX in lipids (first shell, *yellow*; second shell, *light orange*; beyond the second shell, *dark orange*). Each panel shows plots for increasing pressures at a given temperature. Data points are the average among three replicas for DMPC (a single replica was simulated for Δ9-cis-PC), with error bars corresponding to the SD among these replicas. (*b*) Bilayer thickness distributions as a function of temperature and pressure. Color codes are the same as above. Note that liquid bilayers (e.g., at 35°C/1 bar) exhibit one broad peak around 35 Å, whereas gel phases (e.g., at 15°C/2000 bar) are described by a bimodal distribution, with a main peak around 42 Å for the textbook $L_{\beta^{\prime }}$ phase, and a secondary peak at low thickness (≃30 Å) corresponding to interdigitated regions. These spatial distributions were obtained by interpolating the bilayer boundaries on a regular grid, and sampling their spacing (see materials and methods). (*c*) Molecular rendering of the organization of a gel-phase (15°C, 2 kbar) DMPC bilayer around OmpX. The protein surface is colored in gray and the cartoon representation of the *β*-barrel in purple. To help to visualize interdigitation of lipid acyl chains and the match between the hydrophobic thickness of the bilayer and that of the protein (low thickness region indicated by the arrow in *b*), some acyl chains of the lipids of the first lipid shell are colored in blue (*outer leaflet*) and red (*inner leaflet*). According to Luzzati’s nomenclature (51) L_*α*_ designs a fluid-like crystalline phase that has a lamellar structure and conformationally disordered acyl chains in contrast with the L$\beta^{\prime }$ gel phase that features acyl chains in a more extended conformation.

MD simulations also provide high spatial resolution of protein-induced perturbations of the lipids (Fig. 4). Regardless of the P/T conditions, the first two lipid solvation shells around the protein remain essentially liquid, as judged from their specific *gauche* fraction values, i.e., 30%, which corresponds to approximately four *gauche* conformations per chain (50) (Fig. 4 *a*). Under all P/T conditions except low temperature (15°C) and pressure (1 bar), lipids beyond the second shell show *gauche* fraction values typical of those of a gel state (10% *gauche* conformations, or one per chain), showing that the spatial extent of OmpX influence on lipid dynamics depends on P/T thermodynamics conditions, but also includes the first two solvation shells. Finally, for systems that have gelified in the presence of OmpX, the *gauche* fraction of lipids beyond the second solvation shell is very similar to that of the pure DMPC system. Thus, simulations provide evidence that the roughness of the protein surface cannot easily accommodate lipids in the extended all-*trans* conformations for the first two solvation shells.

Bilayer thickness also strongly depends on the lipid phase. The bilayer is thicker in the gel phase and thinner in the fluid phase. Consequently, the fluid-to-gel transition for the DMPC-only bilayer is reflected by the increase in thickness at 15°C/2000 bar (gel phase) compared with 35°C/1 bar (fluid phase) (see Fig. 4 *b*). In the presence of OmpX, the first two shells are liquid-like (i.e., thinner bilayer), while the third shell is more similar to the lipid distribution without OmpX. A clear distinction emerges between the first and second shells, particularly under high-pressure and low-temperature conditions, where the second shell displays a broader distribution, akin to that of a gel phase. Two additional observations can be made. First, the first shell is thinner than the corresponding liquid phase in the absence of OmpX, suggesting that lipids adapt to accommodate the presence of the protein. Second, the gel phase exhibits a bimodal and broad thickness distribution, with a primary peak corresponding to the well-ordered gel phase at large bilayer thickness and a significant population at much smaller thickness values, which can be attributed to interdigitation of the two lipid shells.

We can thus identify two types of perturbation induced by OmpX in DMPC bilayers. The first type is a short-range effect on the first two solvation shells that always remain disordered. This may originate from the roughness of the OmpX transmembrane surface, which would destabilize the extended conformers of the surrounding lipids and induce structural defects; and/or from the necessary hydrophobic matching between the lipid bilayer and the protein transmembrane surface, which would disrupt the organization of the lipids (Fig. 4 *c*). The second type of protein-induced perturbation extends beyond these two shells and shifts the phase transition to higher pressures.

### Correlated structural and dynamic changes in OmpX upon lipid-phase transition

**Figure 5:**
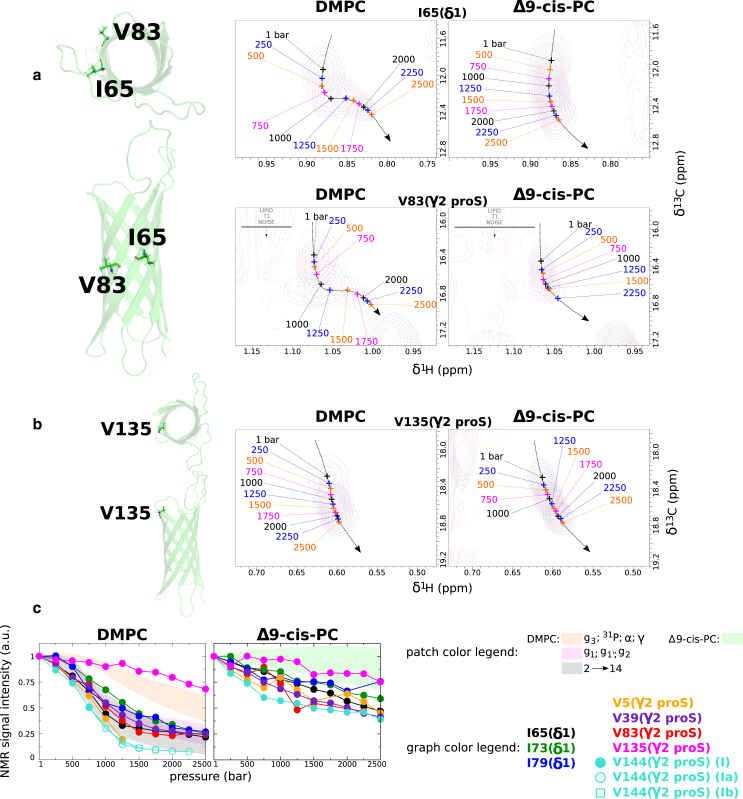
Barotropic evolution of ^1^H NMR signals of membrane-oriented ^13^CH_3_ of OmpX in DMPC and Δ9-cis-PC nanodiscs at 25°C. (*a*) Illustrations of superimposed 2D ^1^H-^13^C SOFAST-HMQC NMR spectra (33) for residues I65 and V83 (see the same illustrations for V5, V39, I73, I79, V144 in Fig. S7; for V144, see also Fig. S13). The numbers represent the hydrostatic pressures that were applied. All spectra are represented on the same intensity scale. The positions of the amino acids are denoted in cartoon representations of OmpX, observed from both a parallel and a perpendicular axis (from the extracellular side) to the plane of the membrane. (*b*) Same as (*a*) for the reference water-exposed residue V135. (*c*) Comparison of the barotropic evolutions of ^13^CH_3_ NMR signal intensity for the membrane-oriented methyls groups and the reference water-exposed V135 either in DMPC (*left*) and Δ9-cis-PC (*right*) at 25°C. The colored patches depict the envelope of the barotropic evolution of intensity for different lipid NMR signals (from Fig. 1*b*). See NMR assignments of ^13^CH_3_-*β*-Ala, ^13^CH_3_-$\delta_{1}$-Ile, and ^13^CH_3_-$\gamma_{2}$ (proS)-Val of OmpX in DMPC and Δ9-cis-PC nanodiscs in Fig. S3 and additional NMR data in Figs. S7–S9 and S13.

**Figure 6:**
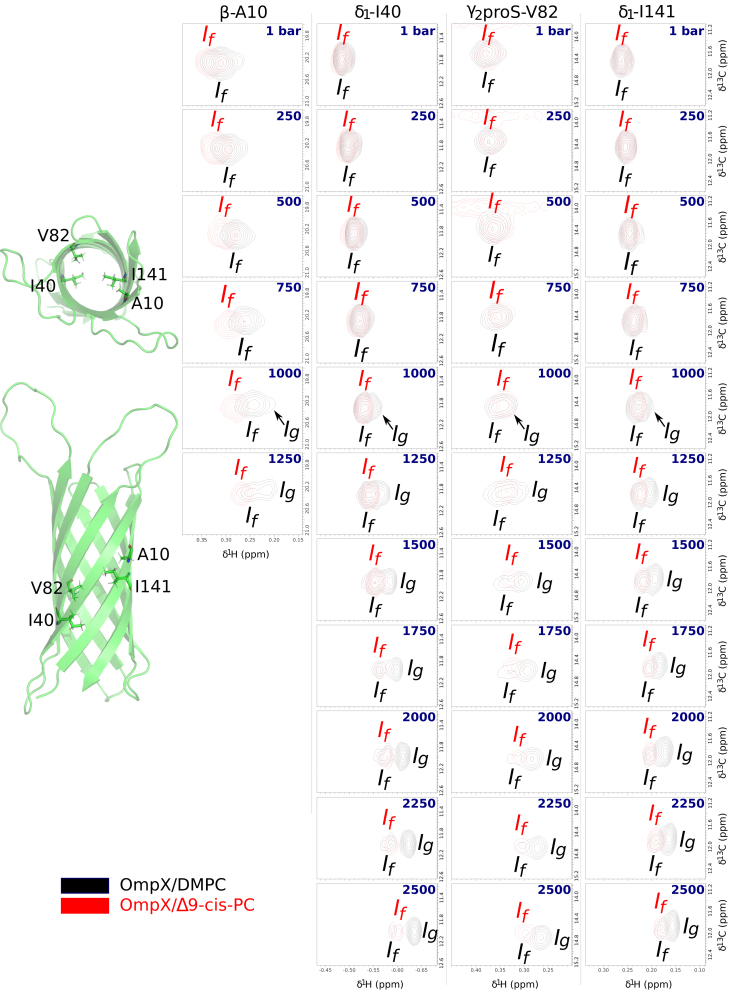
Barotropic evolution of ^1^H NMR signals of barrel interior-oriented ^13^CH_3_ of OmpX in DMPC (in *black*) and Δ9-cis-PC (in *red*) nanodiscs at 25°C (see also Fig. S15). All the spectra are represented at the same scale in both dimensions (i.e., 0.25 and 1.5 ppm in, respectively, the ^1^H and ^13^C dimensions). $I_{f}$ and $I_{g}$ design the protein states when DMPC is in the fluid and gel states, respectively. The numbers in blue correspond to the hydrostatic pressure applied to the NMR sample. On the left are shown a top view (from the extracellular side) and a side view (from an axis parallel to the lipid bilayer) of a cartoon of OmpX (PDB: 2M06 (32)) with the four cavity-oriented residues represented in sticks. See equivalent NMR data collected at 35°C in Fig. S10, putative *χ*1 (N-C*α*-C*β*-C*γ*) rotamers for cavity membrane-oriented I40, V82, and I141 side chains in OmpX crystal structure in Fig. S11 and additional NMR data in Figs. S14 and S15.

**Figure 7:**
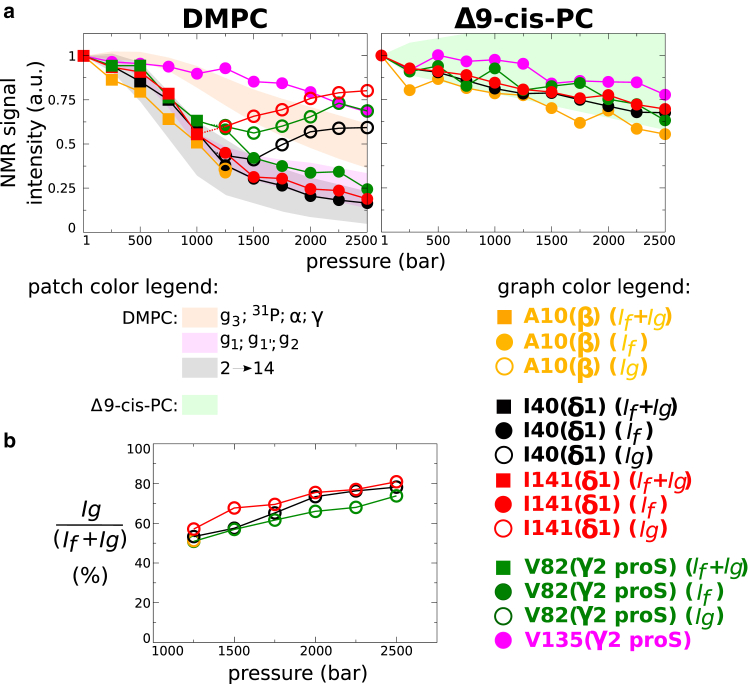
Barotropic evolution of NMR signal intensities of barrel interior-oriented ^13^CH_3_ of OmpX in DMPC and Δ9-cis-PC nanodiscs at 25°C (see also Fig. S15). (*a*) Comparison of the barotropic evolutions of ^13^CH_3_ NMR signal intensity. (*b*) Barotropic evolution of the relative population of $I_{g}$. See same analysis but at 35°C in Fig. S11.

We also monitored OmpX ^1^H and ^13^C NMR signals from the same samples and NMR experiments as (27) used to monitor lipid dynamics. This offered a unique opportunity to assess potential protein conformational changes concomitant to DMPC fluid-gel transition. Thanks to the *β*-barrel geometry of OmpX, we were able to distinguish and compare NMR signals of methyl groups in close contact with the lipid bilayer (residues V5, V39, I65, I73, I79, V83, V144, see Fig. 5), those pointing toward the interior of the cavity (A10, I40, V82, I141, see Figs. 6 and 7), and those located at the extracellular edge of the *β*-barrel (I132, V137). We aimed to determine potential changes that correlate with the DMPC lipid phase transition visible at 25 and 35°C, using Δ9-cis-PC as a negative control. V135, which is located at the apex of the 8th strand and exposed to water, serves as a reliable reference because the barotropic evolution of its NMR signal is very similar in DMPC and Δ9-cis-PC bilayers, showing that V135 is essentially not sensitive to membrane dynamic changes (Fig. S6).

Fig. 5 *a* depicts the barotropic progression, at 25°C, of the NMR signals of the well-resolved membrane-oriented I65-$\delta_{1}$ and V83-$\gamma_{2}$ proS methyls in DMPC and Δ9-cis-PC nanodiscs (I73, I79, V5, V39, and V144 NMR signals are displayed in Fig. S7). Confirming and expanding our previous study (27), we show here that DMPC gelation is associated with a significant decrease in intensity for the methyl groups that are directly exposed to the membrane. This decrease in methyl signal intensity is very similar to that of DMPC acyl chains (Fig. 5 *c*). In contrast, this decrease in intensity for I65-$\delta_{1}$ and V83-$\gamma_{2}$ proS methyls is much reduced in Δ9-cis-PC lipids. This unequivocally shows that the relaxation change of these membrane-oriented methyl groups is directly connected to the changes in dynamics of the neighboring DMPC acyl chains upon phase transition and is not directly due to the applied pressure. The pressure-induced line broadening is most likely due to the gradual constriction of void volumes within this system.

We then analyzed methyl ^1^H and ^13^C chemical shift evolution. Chemical shifts generally report on the local chemical environment including the local protein structure or the organization of water/lipid solvation shells. Figs. 5 *a* and S7 clearly show that these evolutions display a sigmoidal inflection in the DMPC bilayer, whereas peaks shift almost linearly with pressure in the Δ9-cis-PC bilayer. In DMPC, the sigmoidal inflection precisely corresponds to the evolution of the DMPC acyl chain during DMPC phase transition. Note that the variations in carbon chemical shift are very similar between the two lipid compositions. In the proton dimension, however, the difference in chemical shift between 1 and 2500 bar is significantly greater in DMPC than in Δ9-cis-PC, in addition to the aforementioned inflection point. Compared with the data collected with Δ9-cis-PC, this observation is more suggestive of a change in the lipid/protein interface during the phase transition than of structural differences at the protein level. Additional experiments at 15°C (Fig. S8) and 35°C (Fig. S9), and/or using the control Δ9-cis-PC nanodiscs further unambiguously established the coupling between the lipid phase transition in DMPC with both the dynamics, i.e., relaxation, and the chemical environment, i.e., chemical shifts of membrane-oriented methyl groups.

Figs. 6 and 7 are the counterparts of Fig. 5 for amino acid side chains pointing toward the interior of the *β*-barrel. Remarkably, the barotropic evolution of the NMR signal of all well-resolved peaks (A10, I40, V82, I141) is also strongly sensitive to the DMPC main phase transition, despite the absence of direct contact with the lipids. Indeed, an additional NMR signal ($I_{g}$) emerges gradually after the midpoint (around 750–1000 bar) of the lipid phase transition at 25°C (Fig. 6), while the barotropic evolution of the intensity of the initial signal observed at ambient pressure matches those corresponding to acyl chain protons of DMPC (Fig. 7 *a*). At pressures higher than 2000 bar, $I_{g}$ is the predominant state (Figs. 6 and 7 *b*).

These observations strongly suggest that the methyl groups inside the cavity of the *β*-barrel feel, at least partially, the state of the lipid phase in concert with $I_{f}$ and $I_{g}$, corresponding to OmpX experiencing essentially a fluid and a gel phase, respectively. The emergence of $I_{g}$ at a higher pressure (∼1250 bar) when temperature is increased to 35°C is further evidence of this concerted lipid and protein behavior (Figs. S10 and S11). As expected, negative controls with Δ9-cis-PC showed no signal splitting (Fig. 6), confirming that pressure alone is not sufficient to induce the emergence of $I_{g}$ signals. Comparison of methyl peak intensities at 1 and 2500 bar (Fig. 7 *a*) in DMPC suggests that the major factor governing peak intensity changes upon gelation of DMPC is $I_{f}\to I_{g}$ population transfer, related to the DMPC fluid → gel process, and not to changes in dynamics/relaxation due to gelation (which is the dominant process for membrane-oriented methyls). The similarity of methyl ^13^C chemical shifts for $I_{f}$ and $I_{g}$ implies a similar $\chi_{2}$ dihedral angle (52,53), so the observed Δ*δ*^1^H is due to the structural rearrangement of either the same methyl group, e.g., with a different $\chi_{1}$ dihedral angle and/or of the amino acids located in the vicinity of these methyl groups within OmpX interior. The OmpX crystal structure (28) shows that the side chains of the three residues I40, V82, and I141 can adopt at least two different rotamers in the cavity (Fig. S12), allowing them to adjust their structure to changes in the physical properties of the bilayer. The very similar relative population changes with pressure (Fig. 7 *b*) and the structural proximity for I40, I141, and V82 further strongly suggest a concerted conformational change along the *β*-barrel.

Our NMR analysis definitively demonstrates the allosteric protein conformational change upon DMPC gelation induced either by high pressure or low temperature. We observed only one NMR signal for each lipid atom, suggesting fast exchange at the ^1^H chemical shift regime (faster than millisecond timescale) between all chemical environments explored by the lipids, including the different phases and contacts with lipoprotein and IMP. In contrast, the exchange between $I_{f}$ and $I_{g}$ states appears to be at the slow exchange timescale (slower than millisecond timescale), suggesting thermodynamically (partially) coupled but kinetically uncoupled processes between lipid phase behavior and protein conformational change in the cavity. The uncoupled kinetics most likely reflect an allosteric pathway connecting lipids and OmpX core involving intermediate states connected through high energy barriers. This is in accordance with our MD simulations. Indeed, although the lipid-phase transitions were easily detectable in simulations, attempts to detect protein conformational changes failed even if enhanced sampling MD techniques along the relevant dihedral degrees of freedom were used. This indicates that the observed conformational changes in the protein core most likely occur at much slower timescales.

V144 $\gamma_{2}$-proS methyl, which is located at the periplasmic edge of the 8th *β*-strand, is exposed to the bilayer based on our MD simulations. It also explores an additional conformation, $I_{a}$, at the slow timescale, when lipids are in the gel phase (Fig. S13). This strongly suggests that V144 dynamics is connected kinetically to the conformational change with the protein core. The side chains from residues I132 and V137 are spatially close to each other at the two extremities of the extracellular $\beta_{7}$-$\beta_{8}$ strands at the edge of the bilayer and show an even more complex behavior. These methyls explore at 25°C two distinct conformations *I* and II at the slow chemical shift timescale, with state II being disfavored at high pressure, irrespective of the lipid phase. On top of this, state *I*, but not state II, further splits into two additional states $I_{a}$ and $I_{b}$ when DMPC transitions to the gel, with $I_{a}$ being the predominant state at higher pressures (Fig. S14). This reveals a complex conformational landscape, partially dependent on membrane dynamics at the $\beta_{7}$-$\beta_{8}$/loop L4 boundary, which contains several residues related to bacterial virulence (28) (Fig. S3).

## Discussion

Membranes of living cells are typically crowded by integral and peripheral membrane proteins segregating in areas of various lipid and/or protein compositions (20). Each area has distinct membrane physical properties such as fluidity or thickness, and functions. As discussed in (12), gel-like membrane domains *are acceptable for life*, as several recent evidences indicate the coexistence of gel domains with fluid membrane domains (e.g., (54,55,56)). This point highlights the importance of being able to study both lipids and MPs on either side of the main liquid-to-gel phase transition.

In this study, we systematically describe the allosteric dialog between an IMP and its surrounding lipids. Lipid nanodiscs are an ideal tool for this purpose, solvating IMPs with just a few layers of lipids. This prevents any exchange between the boundary lipids and the bulk, thereby preserving the memory of the interaction, as in a membrane, the rate of exchange of lipid molecules between the annular shell and the bulk phase is fast (57). Nanodiscs are compatible with high-resolution NMR data acquisition at the molecular scale (58) on both the lipid and the IMP sides in a single experiment. These nanometric bilayers can transition thermotropically from fluid to gel, but with much lower cooperativity than vesicles, and this cooperativity increases with bilayer size. The transition temperature $T_{m}$ also depends on the size of the nanodisc due to the lipoprotein rim that affects the ordering of lipids; that is, the smaller the nanodisc, the lower the $T_{m}$ (48). It is therefore plausible that different nanodiscs may have distinct pressure response, although this remains to be investigated in greater details. We demonstrated with various lipid compositions that nanometric lipid bilayers can also undergo a gel-to-fluid phase transition at pressures that are comparable or even identical to those observed in much larger systems such as liposomes (27). The utilization of hydrostatic pressure under constant temperature offers a significant advantage by solely taking into account variations in the average volume. In contrast, in isobaric experiments, changing temperature makes it difficult to disentangle kinetic energy from volume effects (59,60,61). From pioneering studies (62), equipment for NMR measurements at high pressure has become readily accessible and user-friendly (cf. materials and methods). Another advantage of combining solution-state NMR with pressure as a thermodynamic variable lies in its potential to explore the gel phase of phospholipids, including those that contain unsaturations, which typically result in negative transition temperatures.

This study builds upon a previous study published in 2022 (27) by highlighting the presence of a phase transition in the presence of the OmpX protein, which was not unambiguously observed in the previous study. The contribution of molecular modeling in the present study also provides an atomic-level view of the behavior of the first lipid layers around OmpX, which is impossible to achieve with NMR, even at very high magnetic fields. NMR only provides an averaged view of the lipids as a whole due to the low resolution of the lipid signals. The complementary negative controls with Δ9-cis-PC are also a crucial part of this study. Finally we reanalyzed the same NMR spectra to highlight the previously unobserved conformational change in OmpX upon lipid phase transition. Here, we show that by introducing a defect in the bilayer organization, OmpX locally perturbs lipids over ∼2 lipid shells. As a result, the phase transition from liquid to gel is shifted to high pressure (e.g., ∼600 bar at 35°C) (Fig. 1). This effect is observed in both experimental and simulated data, and OmpX increases the fluidity of neighboring lipids. This is explained by the tendency of lipids to adapt to the protein surface roughness and the matching of bilayer thickness to the IMP hydrophobic surface.

Our work provides quantitative evidence of the allosteric coupling of lipid dynamics with subtle conformational changes along the transmembrane region of the protein, including in its interior, i.e., away from the lipid bilayer. Amino acid side chains in contact with the lipid bilayer undergo a concerted change in their chemical environment (through their ^1^H NMR chemical shifts) and dynamics (through their ^1^H NMR signal intensities) with the lipid main fluid-to-gel phase transition (Figs. 5 and S7). Astonishingly, we observe also a similar trend for side chains pointing into the cavity of the *β*-barrel with the exception that two states can be distinguished, one favored by a fluid membrane and the other by a gelled bilayer (Figs. 6, 7, and S15). Although the use of ^13^C-labeled, protonated methyl groups immersed within a perdeuterated protein represents the best possible isotopic labeling scheme ever for studying large objects by NMR (63), a quick look at some barotropic evolutions of ^1^H^*N*^-^1^H^*N*^ NMR correlation signals also suggests that the *β*-barrel backbone is sensitive to the lipid phase transition (Fig. S16). However, the sensitivity and the resolution of these signals do not permit as fine an analysis as that with methyls.

Based on the specific *β*-barrel OmpX structure, we propose that the allosteric pathway coupling bulk lipid dynamics with amino acid side chains pointing at the protein core involves minute conformational and dynamic changes of the backbone and H-bond networks. *β*-barrel structures being rather rigid, such conformational changes might involve high energy barriers, explaining the observed slow kinetics. As a consequence, kinetically uncoupled processes between a fast exchange of the lipid phase behavior (faster than millisecond timescale) and slow protein conformational change in the cavity (slower than millisecond timescale), MD simulations with enhanced sampling of side-chain degrees of freedom exhibited a large variance across multiple replicas, highlighting that the conformational landscape of the side chains is influenced by the relaxation of slow (unknown) degrees of freedom in the surrounding environment. Addressing this challenge more accurately will require further methodological developments and investigations.

## Conclusion

Given the well-known impact of membrane composition in IMP function (2), these results shed light on a potential mechanism by which the dynamics of lipids may allosterically control the function of IMP by fine-tuning conformational changes at their binding or active sites, even in the case of extremely rigid IMPs, such as OmpX. We hypothesize that such a mechanism might provide another layer of IMP functional regulation associated with local lipid gelation. G-protein coupled receptors are typical *α*-helical IMP with helical packing varying greatly upon ligand binding in line with their functional activation. Whether membrane collective properties also allosterically alter the G-protein-coupled receptor conformational landscape, beyond specific ligand/lipid binding, still remains to be investigated. Our recent study (27) paves the way to explore such a potential allosteric coupling in *α*-helical IMP. Our approach also opens up a new way of studying a wide variety of fields such as mechanically activated membrane proteins (64,65), the adaptation of biological membranes to pressure in deep-sea organisms (66,67), and drug design (68).

## Acknowledgments

We thank Daniel Picot for his helpful expertize in analyzing the electron density of OmpX with COOT software and Sebastien Billès for his contribution in the assignment of NMR lipid signals. This work was funded by the 10.13039/501100004794Centre National de la Recherche Scientifique (CNRS), 10.13039/501100022108Université de Poitiers, the 10.13039/501100001665Agence Nationale de la Recherche (ANR-17-CE11-0011 and ANR-22-CE29-0020), Laboratoire d’Excellence (LabEx) DYNAMO (ANR-11-LABX-0011), and Equipements d’Excellence (EQUIPEX) CACSICE (ANR-11-EQPX-0008) from the French Ministry of Research. Financial support from the IR INFRANALYTICS FR2054 10.13039/501100004794CNRS for access to NMR spectrometers is gratefully acknowledged. This work was also supported by the 10.13039/501100011658French Infrastructure for Integrated Structural Biology (FRISBI) ANR-10-INBS-0005.

## Author contributions

G.S., J.H., E.L., and L.J.C. conceived of the project. A.P., E.P., C.L.B., and K.M. performed biochemistry. A.P. and L.J.C. performed the NMR sample preparation. C.F., A.P., F.G., E.L., and L.J.C. performed the NMR data collection and analysis. Y.G., J.H., and G.S. performed and analyzed the MD simulations. All authors discussed the data and their interpretation and edited the paper.

## Declaration of interests

The authors declare no competing interests.

## Supporting material

Document S1. Figures S1–S16

Document S2. Article plus supporting material
