# Supplementary material for: Allosteric coupling between a lipid bilayer and a membrane protein
Source: Biophys J. 2025 Jun 27;124(16):2613–26. doi: 10.1016/j.bpj.2025.06.033 (PMC12414673; doi:10.1016/j.bpj.2025.06.033)
Supplement: Document S1. Figures S1–S16 [file mmc1.pdf]

Biophysical Journal, Volume *124*

Supplemental information

Allosteric coupling between a lipid bilayer and a membrane protein

Clarisse Fourel, Yanna Gautier, Alexandre Pozza, François Giraud, Elodie Point, Christel Le Bon, Karine Moncoq, Guillaume Stirnemann, Jérôme Hénin, Ewen Lescop, and Laurent J. Catoire

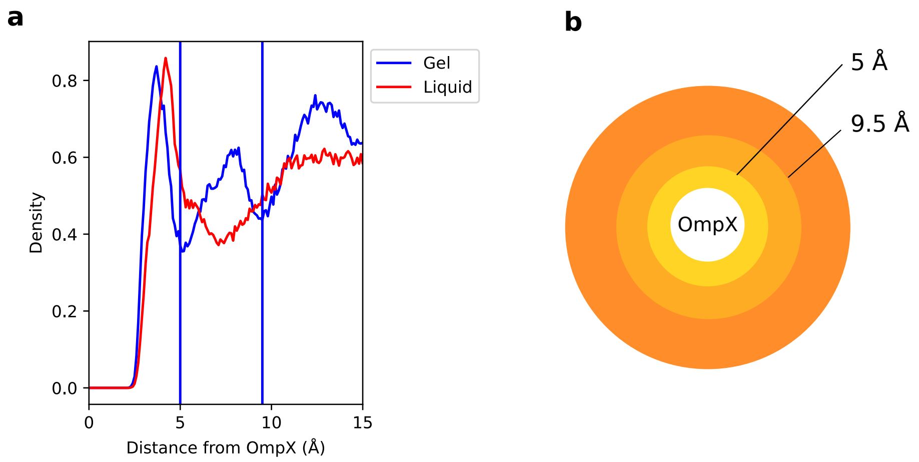

Figure S1. Spatial organization of the lipids around OmpX. *(a)* Density distribution of lipids as a function of distance from the protein OmpX. Conditions (P,T) are 2 kbar and 308 K for the gel phase and 1 bar and 308 K for the fluid phase. *(b)* The organization in layers of lipids around the protein is represented by three circles of different colors, ranging from yellow for the first lipid layer to dark orange for the third layer of lipids, also called bulk lipids, with the second layer of lipids in between. Values of 5 Å and 9.5 Å correspond to the limits of the layers found in plot *(a)*.

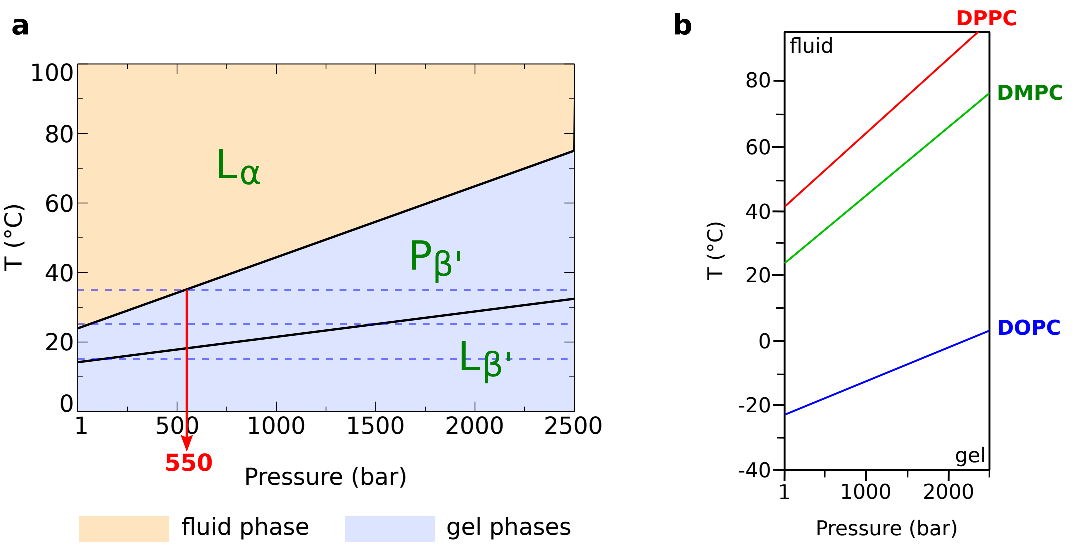

Figure S2. Temperature/Pressure (T/P) phase properties of various lipid vesicles in excess water (in the absence of proteins). (*a*) T/P phase diagram of hydrated multilamellar vesicles of DMPC. In the present study, the pressure ramps have been carried out along three isotherms (15, 25 and 35°C) represented by dashed blue lines. The fluid-like crystalline phase is referred to as L_α according to Luzzati's nomenclature (1). It has a lamellar structure with conformationally disordered acyl chains. Additionally, two solid-like gel phases, $P_{\beta'}$ and $L_{\beta'}$, exist wherein the chains exhibit a more extended conformation. $P_{\beta'}$, commonly referred to as the ripple gel phase, is characterized by a periodic undulation of the bilayer within the lamellae plane. In this phase, the acyl chains are in a higher degree of ordering compared to the L_α phase, and they are tilted in relation to the normal to the bilayer plane. This phase occurs in bilayers consisting of phospholipids with saturated hydrocarbon chains (2), wherein they arrange themselves into a regular hexagonal lattice (3, 4). $L_{\beta'}$ exhibits a lamellar bilayer structure characterized by fully extended and tilted hydrocarbon chains, but it is packed in a slightly distorted hexagonal lattice in comparison to $P_{\beta'}$ (3, 4). The gel-to-gel $P_{\beta'} \rightarrow L_{\beta'}$ pre-transition, which was predicted to happen at approximately 1500 bar at 25°C (5), was not observed in the present study possibly because the periodic length of the ripple phase is ~145 Å (2, 6), i.e., larger than the diameter of the MSP1D1 nanodisc (~10 nm). It is also possible that the similar relaxation properties in the $P_{\beta'}$ and $L_{\beta'}$ phases prevent any observable transition (this diagram is adapted from (5)). (*b*) Fluid and gel phase demarcation lines for DPPC, DMPC and DOPC (based on an abundant literature). No such data is available for Δ9–cis–PC which is believed to remain in a fluid phase in the T/P conditions used in the present study using nanodiscs. This is due to the two cis-double bonds that introduce kinks and make it more difficult to align the fatty chains with each other reducing the ordering effect of pressure. Dipalmitoylphosphatidylcholine (DOPC), in which each acyl chain contains an additional 4 carbon atoms (18:1(Δ9–cis–PC)), has a T_m = -23°C and ~0°C at 1 and 2500 bar (7), respectively (blue line in the diagram). Based on these values, Δ9–cis–PC has necessarily lower T_m due also to shorter fatty chains, by analogy with fully saturated DPPC (16 carbon atoms per acyl chain) (red line) and DMPC (green line) which have T_m equal to 41 and 24°C at 1 bar, respectively.

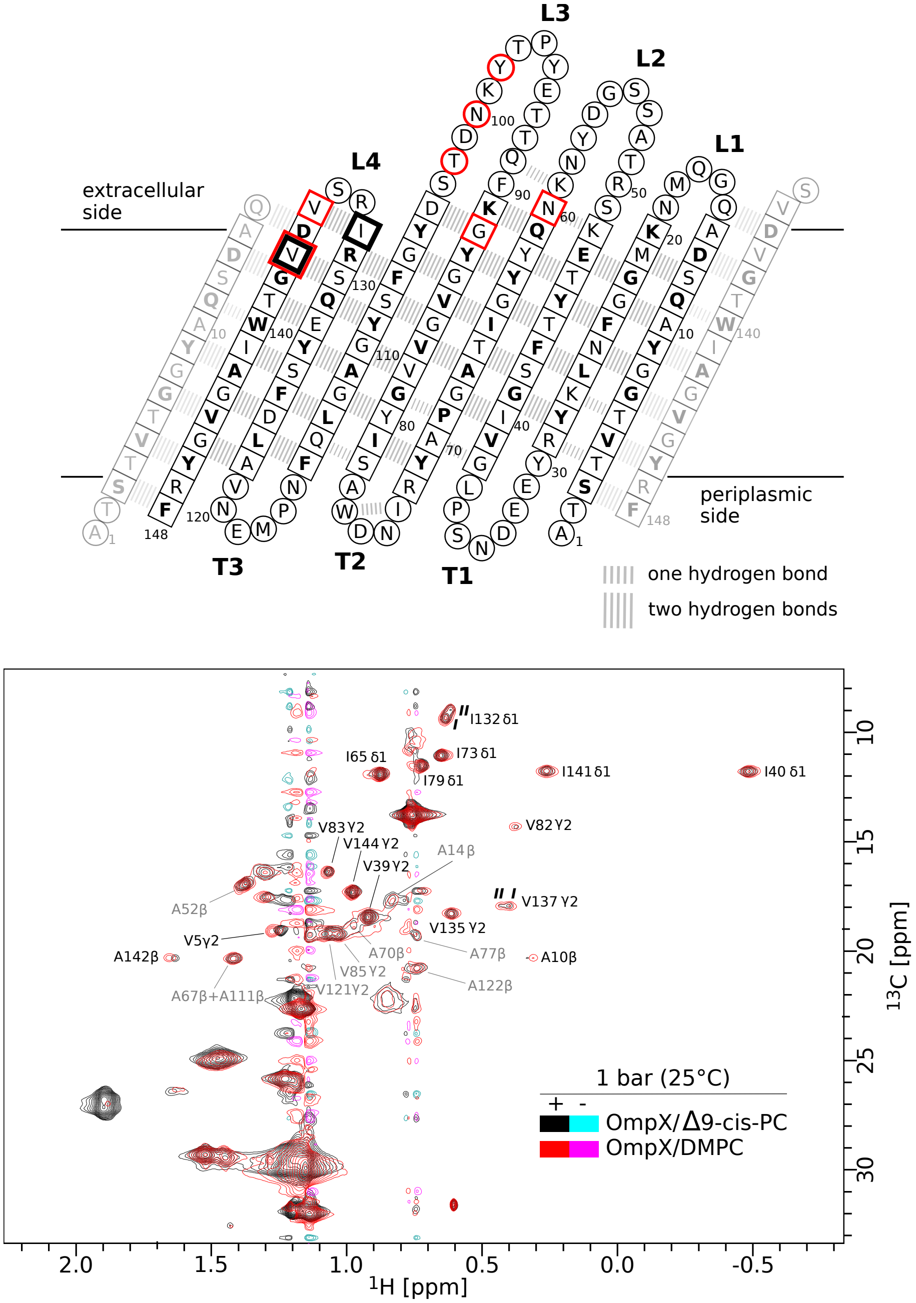

Figure S3. NMR assignments of $^{13}CH_3$–β–Ala, $^{13}CH_3$–δ_1–Ile and $^{13}CH_3$–γ_2(proS)–Val of OmpX in DMPC and Δ9–cis–PC nanodiscs. (*Top*) Snake diagram of OmpX. Residues in β–strands are shown in squares, the others in circle. I132 and V137 that display splitted signals are framed with a bold square. Residues of the β-barrel that point their side chains to the lipid bilayer are written in bold black letters. The hydrogen bond network is based on OmpX NMR structure in MSP1D1 nanodiscs (PDB ID: 1QJ8 (8)). **L** and **T** stands for extracellular loops and periplasmic turns, respectively. Residues involved in virulence and defense of the analog of OmpX protein Ail in *Y. enterocolitica* are squared/circled in red (from Vogt and Schulz (9)). (*Bottom*) Two superimposed 2D ^{1}H,^{13}C SOFAST-HMQC (10) spectra collected at ambient pressure (1 bar) and 25°C. Residues labeled in grey were not taken into consideration due to either spectral crowding or lipid noise signals. The unlabeled signals correspond to lipid and lipoprotein CH_n moieties as described in Fig. S4.

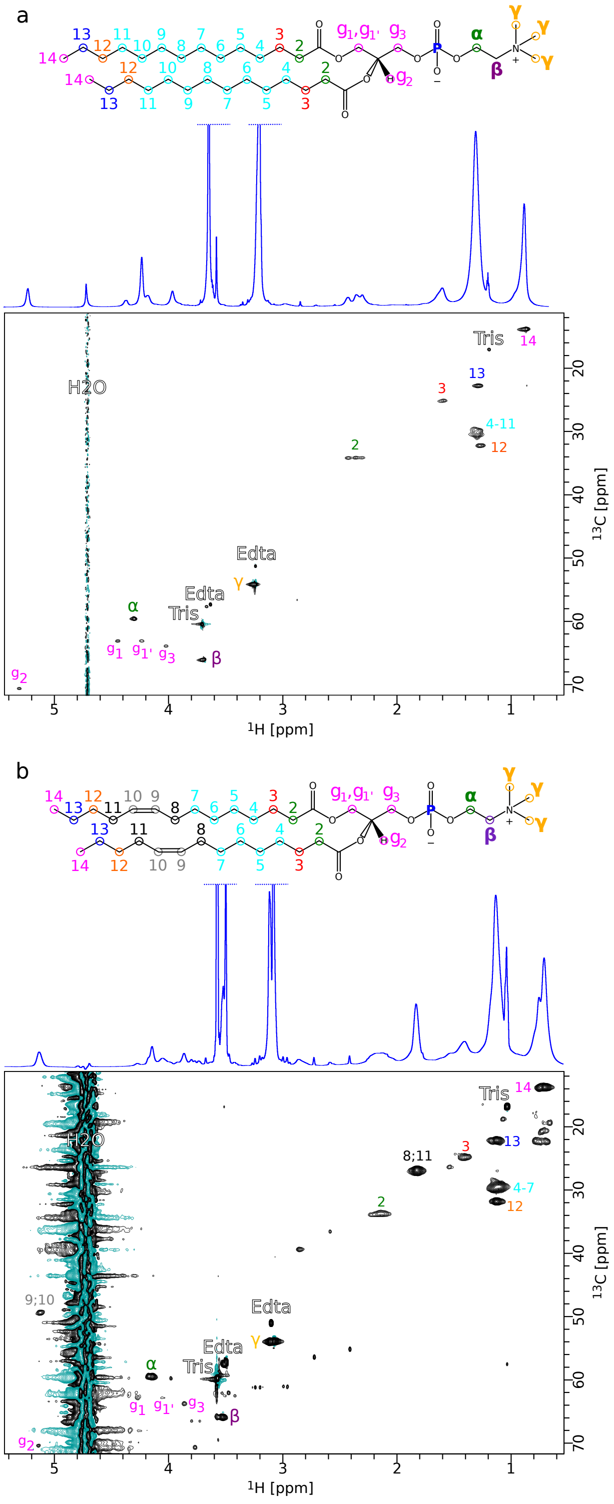

Figure S4. ^{1}H,^{13}C NMR assignments of DMPC (*top*) and Δ9–cis–PC (*bottom*) CH_n in OmpX-devoid nanodiscs. On top of each 2D ^{1}H,^{13}C SOFAST-HMQC (10) spectrum is displayed a 1D ^{1}H spectrum and the lipid chemical structure with a color code which is reproduced on the assignments. The assignments are based on 2D ^{1}H,^{1}H TOCSY, NOESY and COSY experiments. The unlabeled signals correspond to the lipoprotein MSP1D1.

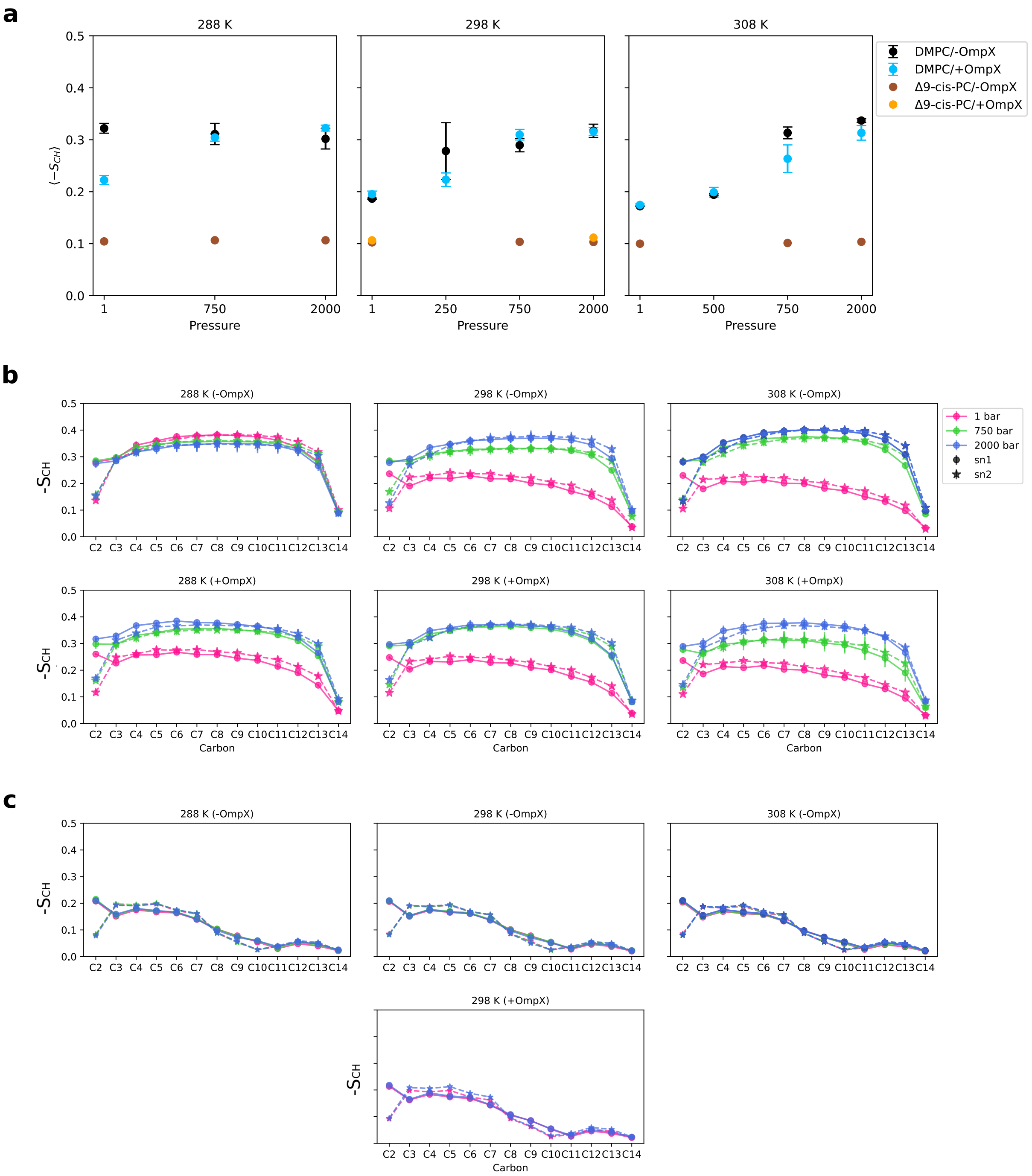

Figure S5. Local order parameters calculated from MD simulations. *(a)* Order parameters of the lipid tails averaged across both tails and across the entire lipid population on the last 300 ns of the trajectory for DMPC and Δ9-cis-PC lipids, with and without OmpX in the lipid bilayer. The order parameters as a function of carbon number in the lipid tails for *(b)* DMPC and *(c)* Δ9-cis-PC lipids, with and without OmpX embedded in the bilayer. Details for each tail are provided. Values averaged over three replicas (error bars represent standard deviation) are shown. The lipid order parameters S_{CH} were calculated according to the following formula : $S_{CH} = \frac{1}{2}\langle 3cos^2\theta - 1\rangle$, where θ is the angle between the bilayer normal and the C-H bond vector (11). The order parameters were calculated for each lipid and each carbon along the two lipid tails every 0.1 ns on the last 300 ns of the trajectory.

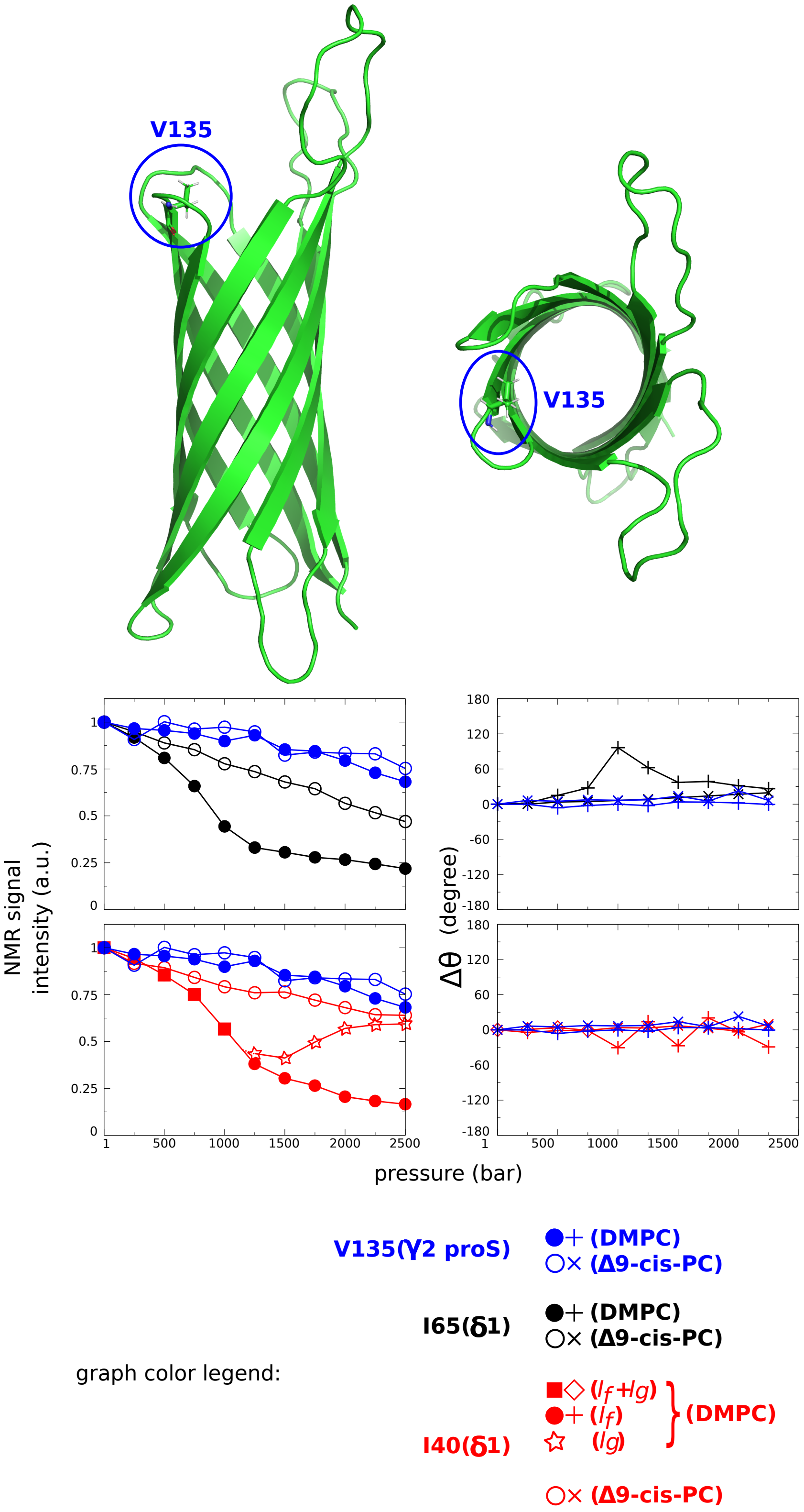

Figure S6. Comparison of V135($\gamma_2$pro*S*) $^{13}CH_3$ barotropic behavior NMR signal with membrane and cavity oriented methyl groups. V135 is located at the apex of the 8th strand and exposed to water. Among all the $^{13}CH_3$ of OmpX studied here, this methyl group represents an ideal reference as its NMR signal has been found to be the less impacted the lipid content, as shown by both the barotropic evolution of its intensity and pressure-dependence in ^{1}H and ^{13}C chemical shifts ($\Delta\theta$). (*Top*) Location of V135 in OmpX. (*Bottom*) Comparison of the barotropic evolution of $^{13}CH_3$ NMR intensities and chemical shifts ($\Delta\theta = \theta(P_i) - \theta(1\text{ bar})$; see the definition of θ on page 14) between V135($\gamma_2$pro*S*) and membrane-oriented I65(δ_1) and cavity-oriented I40(δ_1).

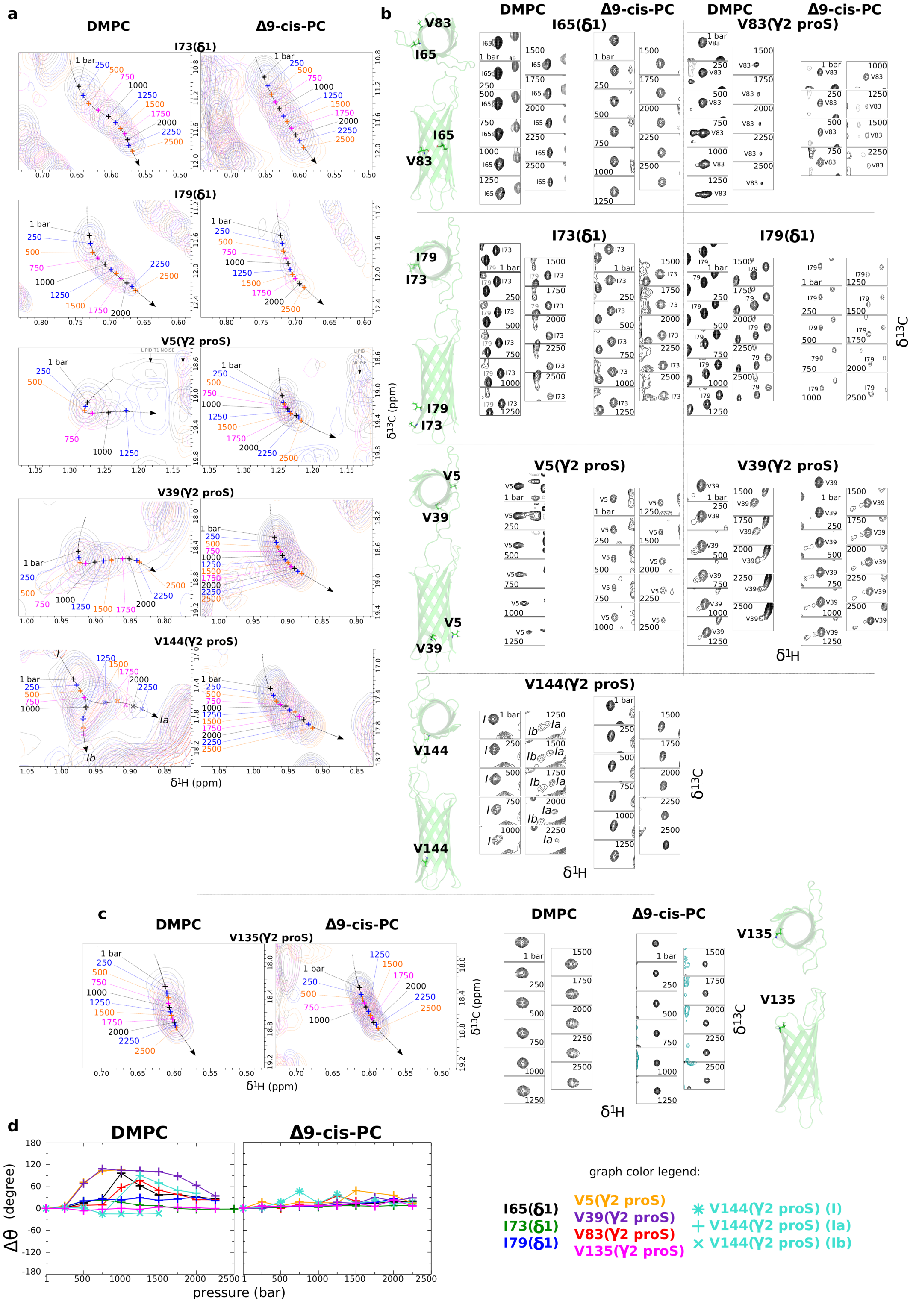

Figure S7. Barotropic evolution of membrane-oriented $^{13}CH_3$ NMR signals of OmpX in DMPC and Δ9–cis–PC nanometric bilayers at 25°C. (*a*) In complement to Fig. 5, Illustrations of superimposed 2D ^{1}H,^{13}C SOFAST-HMQC NMR spectra (10) for residues V5, V39, I73, I79 and V144. The numbers represent the hydrostatic pressures that were applied. All spectra have been represented at the same scale. For V144, *cf.* also Fig. S13. (*b*) Each panel represents successive 2D ^{1}H,^{13}C SOFAST-HMQC spectra acquired along the pressure ramp. They are all represented at the same scale, *i.e.*, spectral widths of 237.5 and 358.2 Hz, respectively in the ^{1}H and ^{13}C dimensions. For each amino acid, these spectra are represented superimposed to each other in (*a*) or Fig. 5a (for I65 and V83). (*c*) Same as *a,b* for the reference residue V135. (*d*) Representations of the barotropic evolution of the $^{13}CH_3$ chemical shift cross peaks through the angle θ ($\Delta\theta = \theta(P_i) - \theta(1\text{ bar})$; see the definition of θ on page 17).

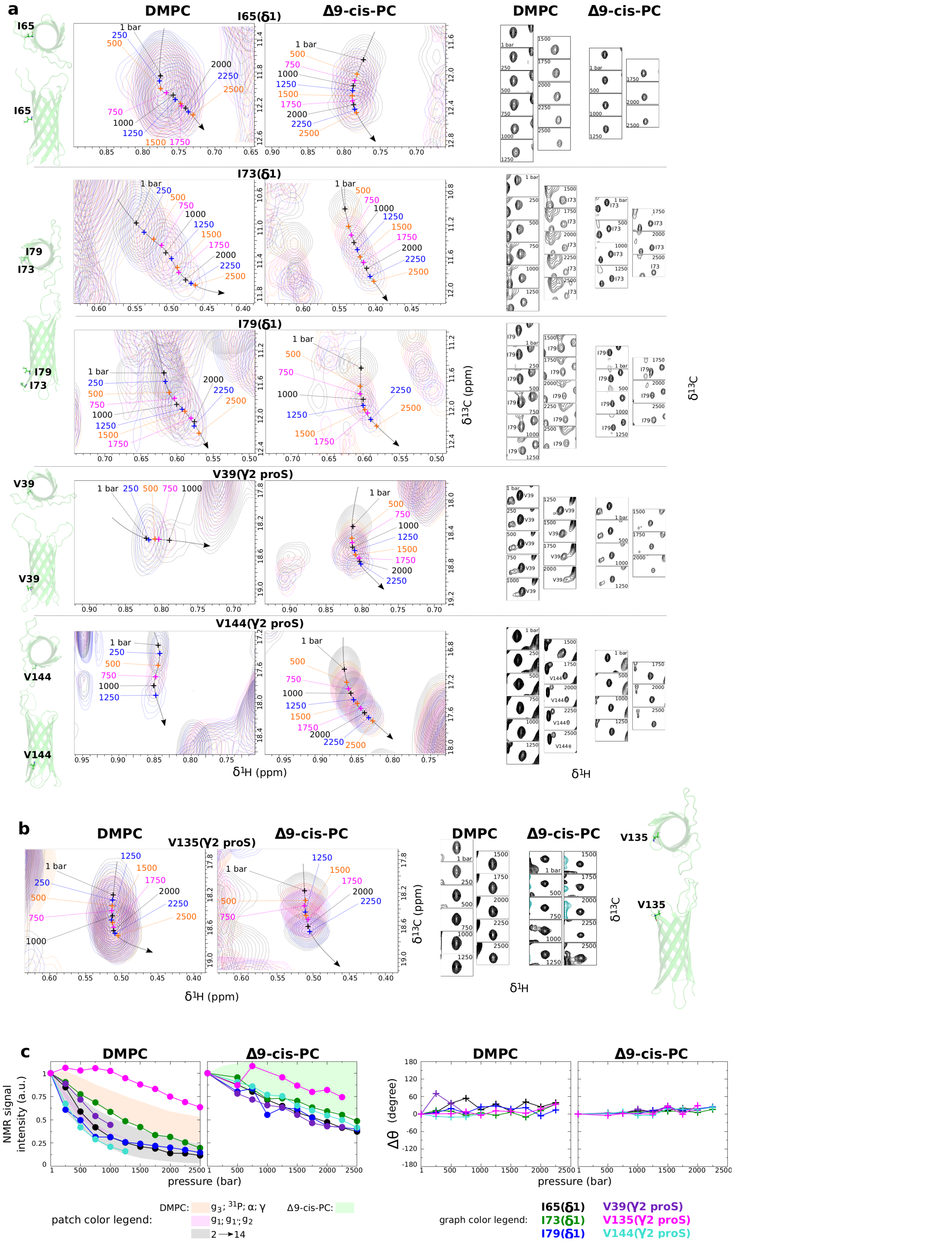

Figure S8. Barotropic evolution of NMR signals of membrane-oriented $^{13}CH_3$ of OmpX in DMPC and Δ9–cis–PC nanodiscs at 15°C. Legend same as Fig. S7 (V5 and V83 where not measured due to to low signal-to-noise ratio in the spectra at 15°C).

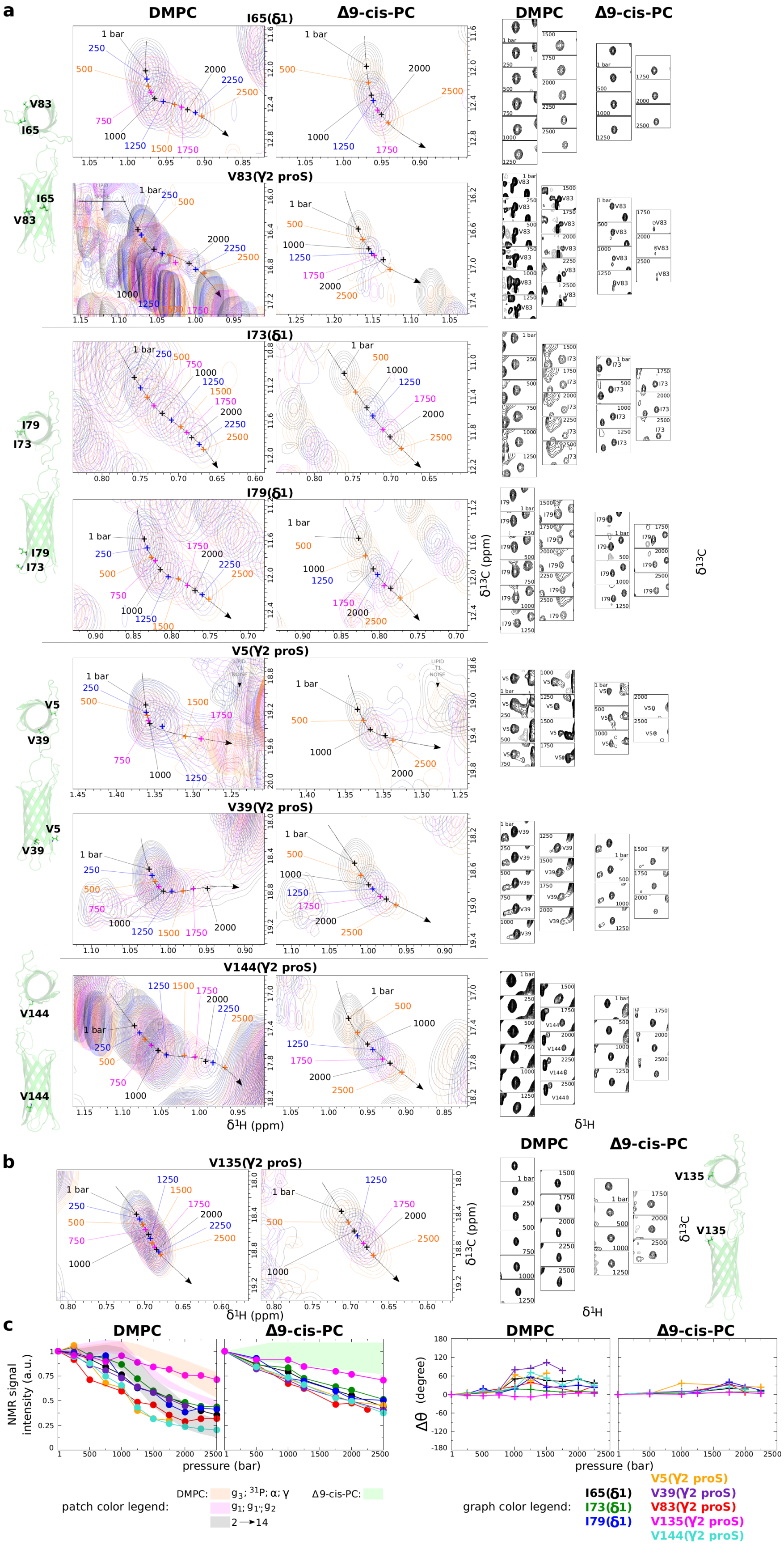

Figure S9. Barotropic evolution of NMR signals of membrane-oriented $^{13}CH_3$ of OmpX in DMPC and Δ9–cis–PC nanodiscs at 35°C. Legend same as Fig. S7.

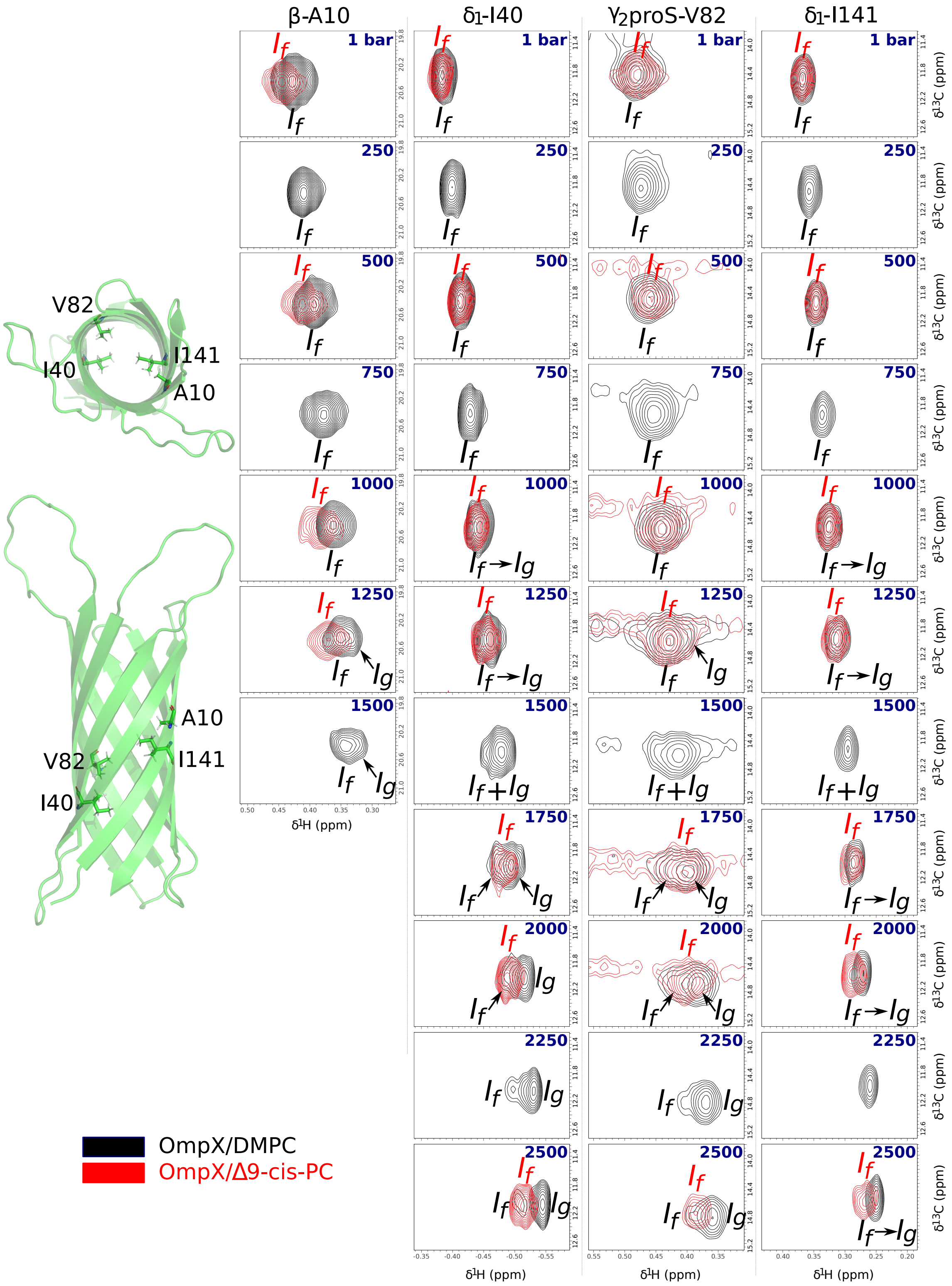

Figure S10. Barotropic evolution of NMR signals of barrel interior-oriented $^{13}CH_3$ of OmpX in DMPC (in black) and Δ9–cis–PC (in red) nanodiscs at 35°C. Legend same as Fig. 6. Compared to data at 25°C (Fig. 6), I141 displays a faster chemical exchange between I_f and I_g. NMR experiments at 250, 750, 1500 and 2250 bar were not performed with OmpX/Δ9–cis–PC nanodiscs.

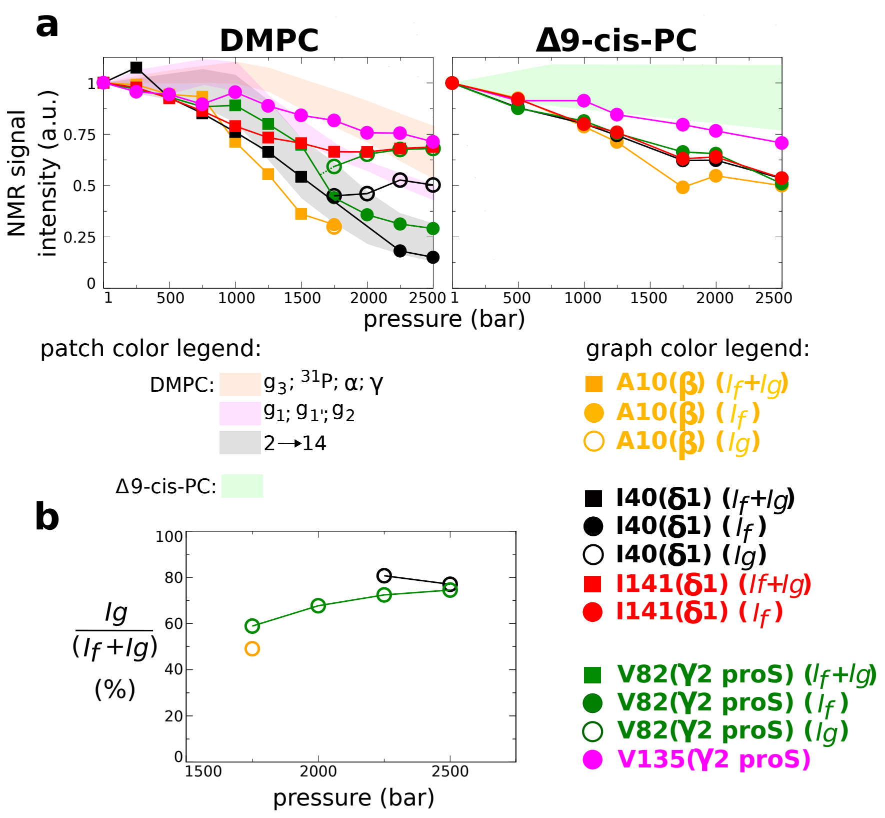

Figure S11. Barotropic evolution of NMR signal intensities of barrel interior-oriented $^{13}CH_3$ of OmpX in DMPC and Δ9–cis–PC nanodiscs at 35°C (in complement to Fig S8). (*a*) Comparison of the barotropic evolutions of $^{13}CH_3$ NMR signal intensity. (*b*) Barotropic evolution of the relative population of I_g.

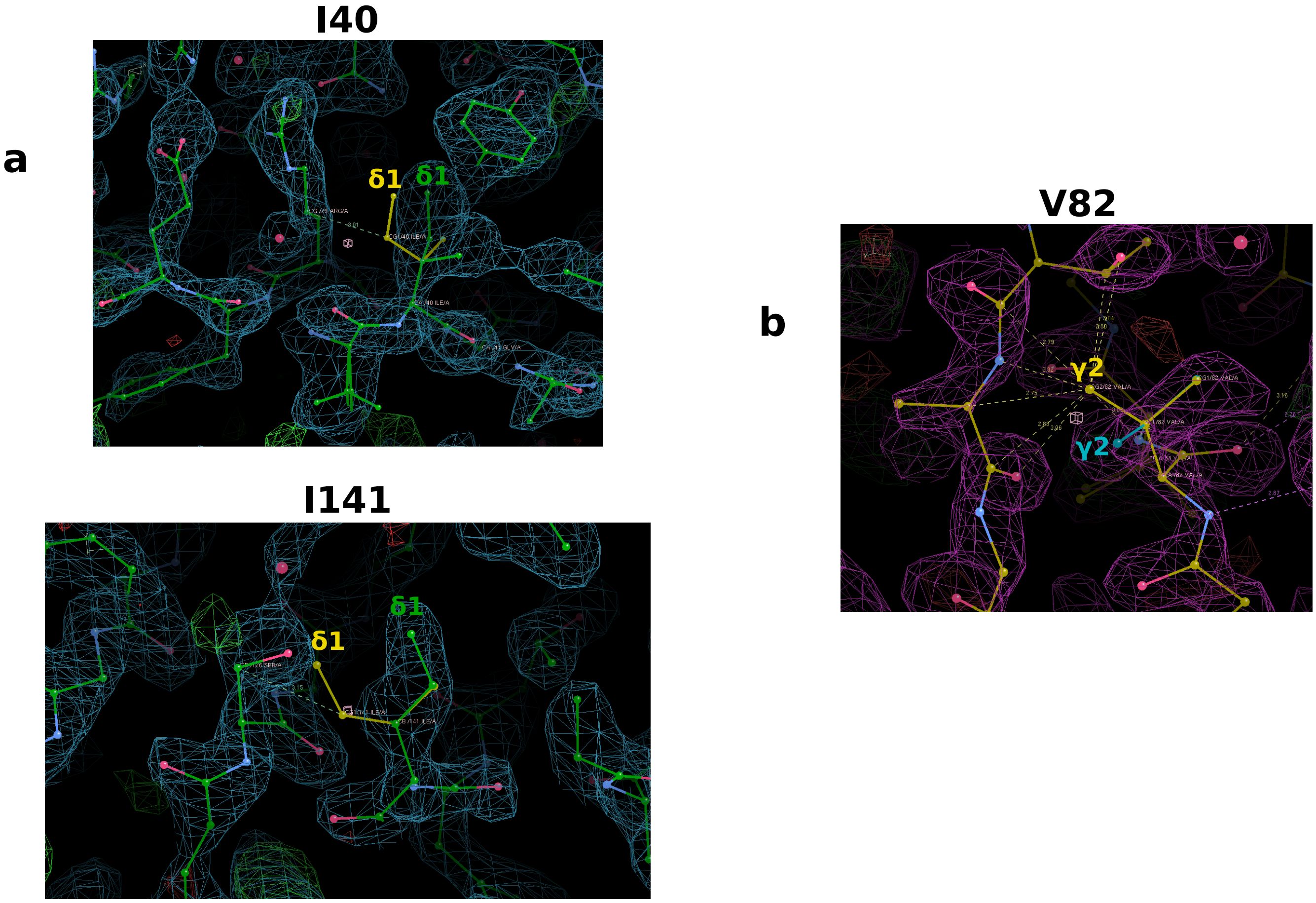

Figure S12. Putative $\chi 1$ (N–Cα–Cβ–Cγ) rotamers for cavity-membrane oriented I40, V82 and I141 side chains.extracellularextracellularextracellular (*a*) close-ups in the I40 (Top) and I141 (bottom) and V82 (*b*) regions. Amino acids are represented in a ball and stick representation. The electron density is represented in *blue* (*a*) or *purple* (*b*) from OmpX crystal structure (PDB ID: 1QJ8). In *green* (*a*) or *blue* (*a*,*b*), the amino acids correspond to the original structure and in *yellow* (*a* and *b*) is represented for each panel one alternative $\chi 1$ rotamer causing no additional clash. A rotamer with a side chain displaying ^{13}C–^{13}C distances $\geq$ 3Å with its neighborhood is considered conceivable. In the case of I141, there is a steric clash with S126 that would require a reorientation of S126 side chain. Representations and rotamer calculation have been generated by *Coot* software (12).

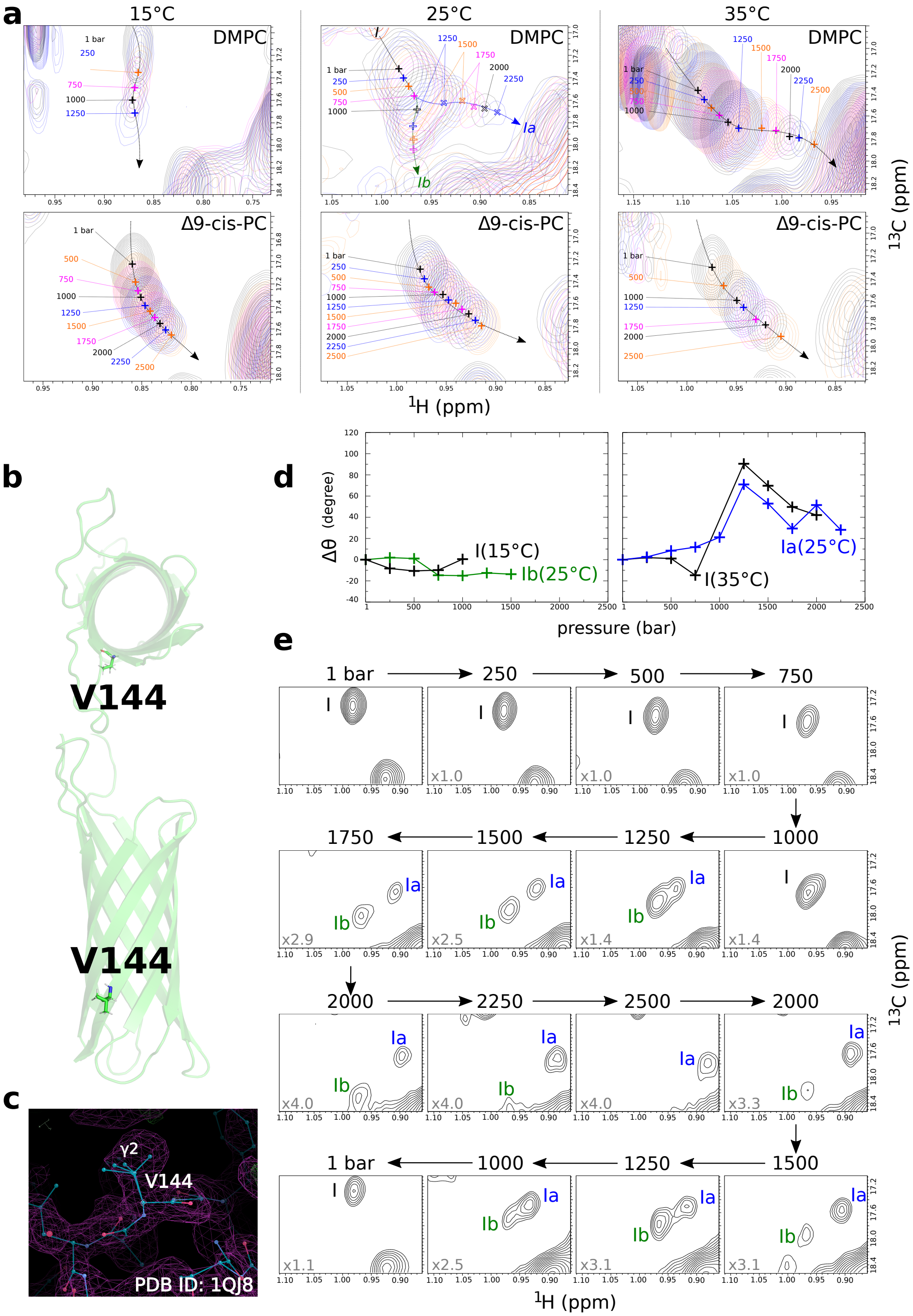

Figure S13. Comparison of the barotropic evolution of γ_2-pro*S*-V144 methyl group observed at 15, 25 and 35°C. (*a*) Superimposed 2D ^{1}H,^{13}C SOFAST-HMQC NMR spectra. The numbers represent the hydrostatic pressures that were applied. All spectra have been represented at the same intensity scale. (*b*) Position of V144 denoted on cartoon representations of OmpX, observed from both a parallel and perpendicular axis (from the extracellular side) to the plane of the membrane. (*c*) Two possible orientations of V144 isopropyl group in OmpX crystal structure represented with balls and sticks in the electron density (PDB ID: 1QJ8). (*d*) Comparison of the pressure-dependence of methyl combined ^{1}H and ^{13}C NMR chemical shifts, $\Delta\theta$, between I(15°C) and I_b(25°C) signals (left) and between I(35°C) and I_a(25°C) (right) (see θ definition on page 17). (*e*) Sequential description of the reversible barotropic evolution of I, I_a and I_b signals at 25°C.

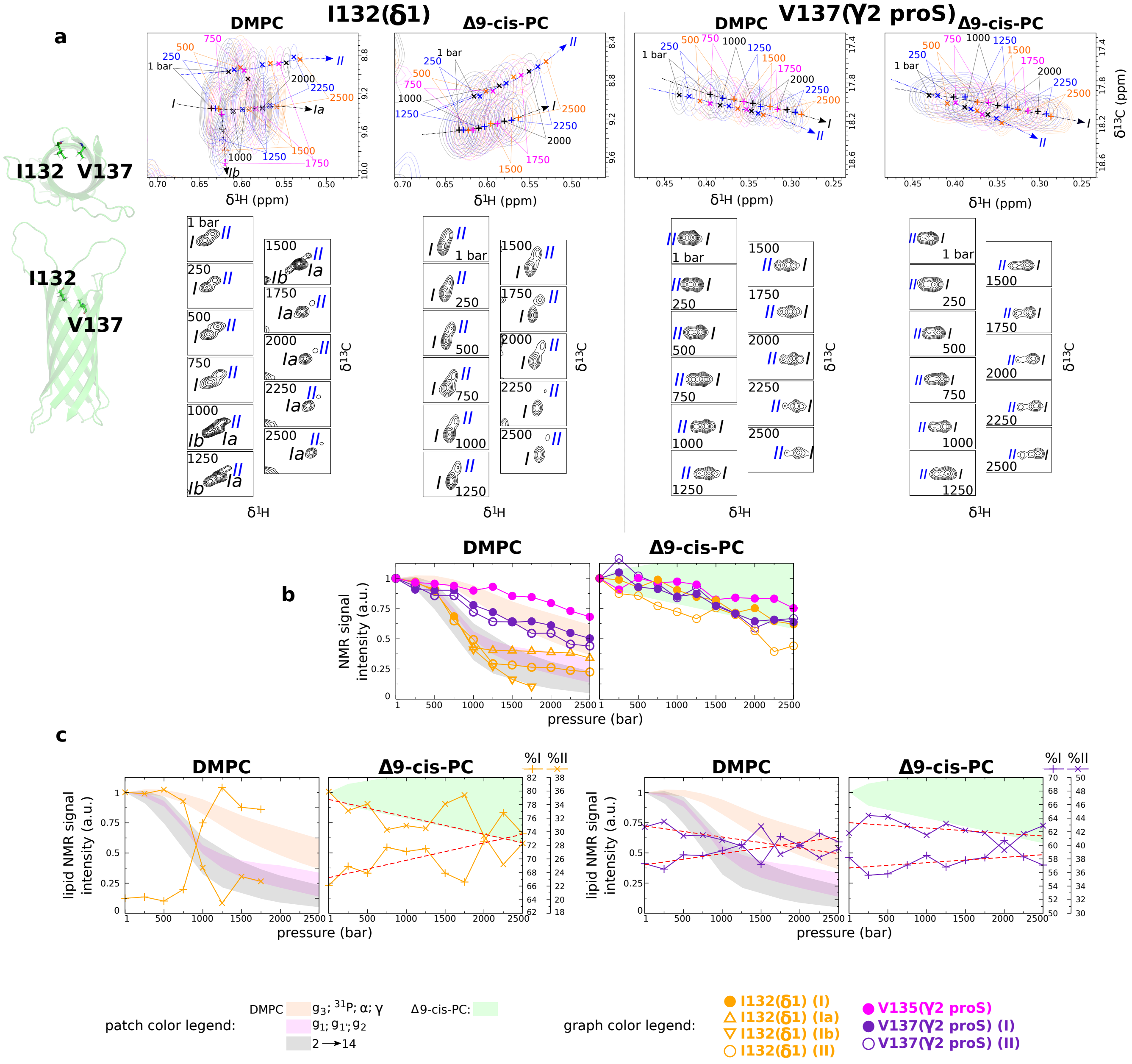

Figure S14. Barotropic evolution of I132(δ_1) and V137($\gamma_2$proS) $^{13}CH_3$ NMR signals of OmpX in DMPC and Δ9–cis–PC nanometric bilayers at 25°C. (*a*) Superimposed 2D ^{1}H,^{13}C SOFAST-HMQC NMR spectra (10) (*Top*) and successive 2D ^{1}H,^{13}C SOFAST-HMQC spectra acquired along the pressure ramp (*Bottom*). All spectra are represented at the same scale, *i.e.*, spectral widths of 237.5 and 358.2 Hz, respectively in the ^{1}H and ^{13}C dimensions. Numbers indicate the pressure applied. (*b*) Evolution of NMR signal intensities under pressurization. (*c*) Evolutionary trajectories of populations *I* and *II* for I132 (left) and V137 (right). The red dashed lines represent linear regression fits.

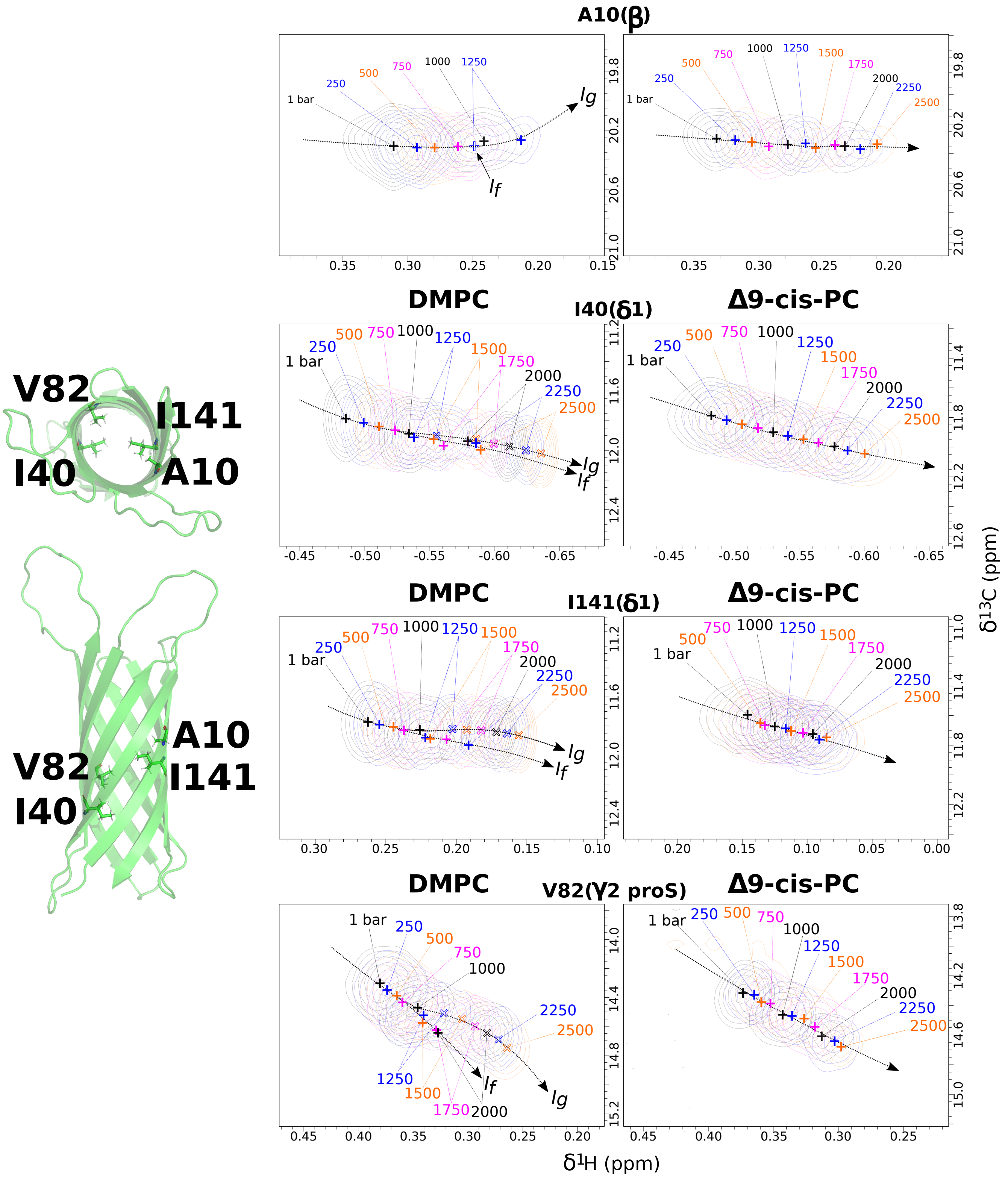

Figure S15. Barotropic evolution of interior cavity-oriented $^{13}CH_3$ NMR signals of OmpX in DMPC and Δ9–cis–PC nanometric bilayers at 25°C (in complement to Fig. 6). Illustrations of superimposed 2D ^1H,^{13}C SOFAST-HMQC NMR spectra (10) for residues A10, I40, V82 and I141. The numbers represent the hydrostatic pressures that were applied. All spectra have been represented at the same scale. I_f and I_g refer to fluid and gel states, respectively (see also data concerning the reference residue V135 in Fig. S7c).

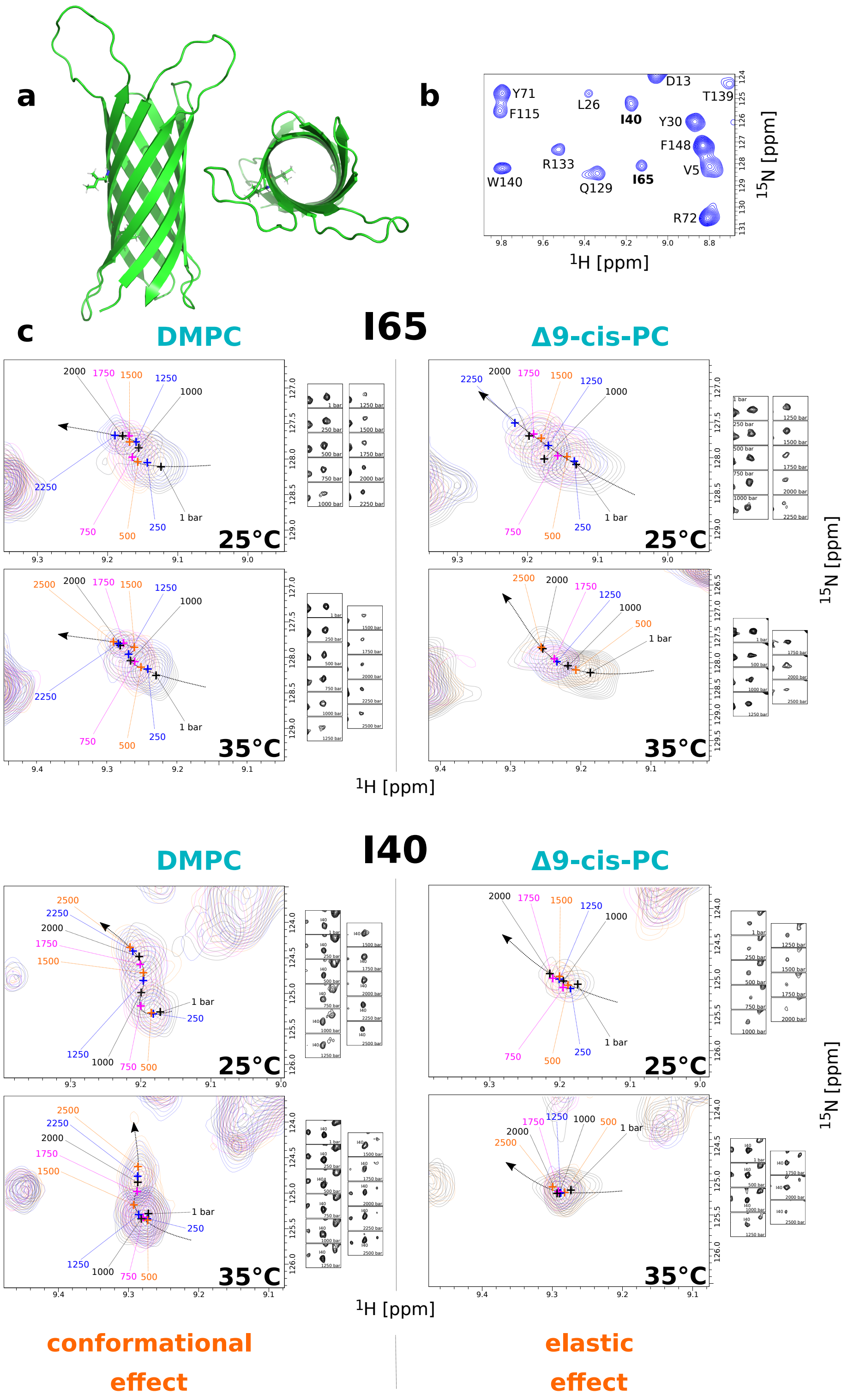

Figure S16. Illustration of barotropic evolution of $^{1}H^{N}$,^{15}N correlation peaks of residues I40 and I65 from 2D ^{1}H,^{15}N SOFAST experiments of OmpX in DMPC (*left*) or Δ9-cis-PC (*right*). All the spectra are represented at the same scale.

Analysis of pressure-induced evolution of OmpX $^{13}CH_3$ chemical shifts: definition of the angle θ.Analysis

To visualize the impact of pressure on the evolution of $^{13}CH_3$ chemical shifts, the angle θ is defined as follows:

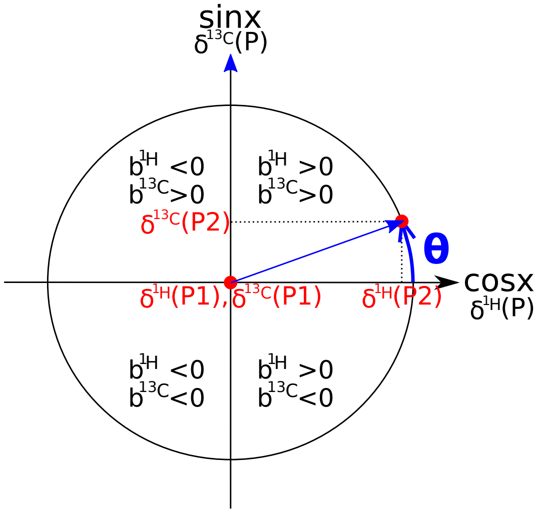

$$cos\theta = \frac{\delta^{1H}(P2)-\delta^{1H}(P1)}{[(\delta^{1H}(P2)-\delta^{1H}(P1))^2+[(\delta^{13C}(P2)-\delta^{13C}(P1))\times\frac{\gamma^{13C}}{\gamma^{1H}}]^2]^{1/2}}$$

$$sin\theta = \frac{[\delta^{13C}(P2)-\delta^{13C}(P1)]\times\frac{\gamma^{13C}}{\gamma^{1H}}}{[(\delta^{1H}(P2)-\delta^{1H}(P1))^2+[(\delta^{13C}(P2)-\delta^{13C}(P1))\times\frac{\gamma^{13C}}{\gamma^{1H}}]^2]^{1/2}}$$

where, $b^{1H} = \frac{\delta^{1H}(P2)-\delta^{1H}(P1)}{\Delta P}$; $b^{13C} = \frac{\delta^{13C}(P2)-\delta^{13C}(P1)}{\Delta P}$

For a given pressure range ($\equiv P2 - P1$), θ contains information on the linear coefficient b of both the proton (b^{1H}) and ^{13}C (b^{13C}). As a first approximation, we consider the b coefficients as an average value in the pressure range $P2 - P1$. In our case, $P2 - P1$ is comprised between 100 and 250 bar.

so,

$$cos\theta = \frac{b^{1H}}{[(b^{1H})^2+(b^{13C}\times\frac{\gamma^{13C}}{\gamma^{1H}})^2]^{1/2}} \text{ or } \frac{b^{1H}}{[(b^{1H})^2+(b^{15N}\times\frac{\gamma^{15N}}{\gamma^{1H}})^2]^{1/2}}$$

$$sin\theta = \frac{b^{13C}\times\frac{\gamma^{13C}}{\gamma^{1H}}}{[(b^{1H})^2+(b^{13C}\times\frac{\gamma^{13C}}{\gamma^{1H}})^2]^{1/2}} \text{ or } \frac{b^{15N}\times\frac{\gamma^{15N}}{\gamma^{1H}}}{[(b^{1H})^2+(b^{15N}\times\frac{\gamma^{15N}}{\gamma^{1H}})^2]^{1/2}}$$

with:

$\gamma^{1H} = 267.52218744\times10^{-6}$ rad×s^{-1}×T^{-1}
and
$\gamma^{13C} = 67.2828\times10^{-6}$ rad×s^{-1}×T^{-1}

⋆ if cosθ >0 (b^{1H} >0) and sinθ >0 (b^{13C} >0):

$$\theta^{13CH_3} = arccos(\frac{b^{1H}}{[(b^{1H})^2+(b^{13C}\times\frac{\gamma^{13C}}{\gamma^{1H}})^2]^{1/2}}) \equiv arcsin(\frac{b^{13C}\times\frac{\gamma^{13C}}{\gamma^{1H}}}{[(b^{1H})^2+(b^{13C}\times\frac{\gamma^{13C}}{\gamma^{1H}})^2]^{1/2}})$$

⋆ if cosθ <0 (b^{1H} <0) and sinθ >0 (b^{13C} >0):

$$\theta^{^{13}CH_3} = arccos(\frac{b^{^1H}}{[(b^{^1H})^2+(b^{^{13}C}\times\frac{\gamma^{13C}}{\gamma^{1H}})^2]^{1/2}}) \equiv \Pi - arcsin(\frac{b^{^{13}C}\times\frac{\gamma^{13C}}{\gamma^{1H}}}{[(b^{^1H})^2+(b^{^{13}C}\times\frac{\gamma^{13C}}{\gamma^{1H}})^2]^{1/2}})$$

⋆ if cosθ <0 ($b^{^1H}$ <0) and sinθ <0 ($b^{^{13}C}$ <0):

$$\theta^{^{13}CH_3} = -arccos(\frac{b^{^1H}}{[(b^{^1H})^2+(b^{^{13}C}\times\frac{\gamma^{13C}}{\gamma^{1H}})^2]^{1/2}}) \equiv -\Pi - arcsin(\frac{b^{^{13}C}\times\frac{\gamma^{13C}}{\gamma^{1H}}}{[(b^{^1H})^2+(b^{^{13}C}\times\frac{\gamma^{13C}}{\gamma^{1H}})^2]^{1/2}})$$

⋆ if cosθ >0 ($b^{^1H}$ >0) and sinθ <0 ($b^{^{13}C}$ <0):

$$\theta^{^{13}CH_3} = -arccos(\frac{b^{^1H}}{[(b^{^1H})^2+(b^{^{13}C}\times\frac{\gamma^{13C}}{\gamma^{1H}})^2]^{1/2}}) \equiv arcsin(\frac{b^{^{13}C}\times\frac{\gamma^{13C}}{\gamma^{1H}}}{[(b^{^1H})^2+(b^{^{13}C}\times\frac{\gamma^{13C}}{\gamma^{1H}})^2]^{1/2}}) \text{ gradually}$$

To facilitate the comparison of θ between different $^{13}CH_3$, we define $\Delta\theta$ in order to have a $\Delta\theta$ equal to 0 at ambient pressure for all methyls:

$$\Delta\theta = \theta(P_i) - \theta(1\text{ bar})$$

REFERENCES

1. Luzzati, V., 1968. X-ray diffraction studies of lipid-water systems. In D. Chapman, editor, Biological Membranes, Academic Press, New York, volume 1, 71–123.

2. Akabori, K., and J. F. Nagle, 2015. Structure of the DMPC lipid bilayer ripple phase. Soft Matter 11:918–926.

3. Tardieu, A., V. Luzzati, and F. C. Reman, 1973. Structure and polymorphism of the hydrocarbon chains of lipids: a study of lecithin-water phases. Journal of Molecular Biology 75:711–733.

4. Janiak, M. J., D. M. Small, and G. G. Shipley, 1976. Nature of the Thermal pretransition of synthetic phospholipids: dimyristolyl- and dipalmitoyllecithin. Biochemistry 15:4575–4580.

5. Al-Ayoubi, S. R., P. K. F. Schinkel, M. Berghaus, M. Herzog, and R. Winter, 2018. Combined effects of osmotic and hydrostatic pressure on multilamellar lipid membranes in the presence of PEG and trehalose. Soft Matter 14:8792–8802.

6. Wack, D. C., and W. W. Webb, 1989. Synchrotron x-ray study of the modulated lamellar phase P beta ' in the lecithin-water system. Physical Review. A 40:2712–2730.

7. Winter, R., and C. Jeworrek, 2009. Effect of pressure on membranes. Soft Matter 5:3157–3173.

8. Hagn, F., M. Etzkorn, T. Raschle, and G. Wagner, 2013. Optimized phospholipid bilayer nanodiscs facilitate high-resolution structure determination of membrane proteins. Journal of the American Chemical Society 135:1919–1925.

9. Vogt, J., and G. E. Schulz, 1999. The structure of the outer membrane protein OmpX from Escherichia coli reveals possible mechanisms of virulence. Structure (London, England: 1993) 7:1301–1309.

10. Amero, C., P. Schanda, M. A. Durá, I. Ayala, D. Marion, B. Franzetti, B. Brutscher, and J. Boisbouvier, 2009. Fast two-dimensional NMR spectroscopy of high molecular weight protein assemblies. Journal of the American Chemical Society 131:3448–3449.

11. Piggot, T. J., J. R. Allison, R. B. Sessions, and J. W. Essex, 2017. On the Calculation of Acyl Chain Order Parameters from Lipid Simulations. Journal of Chemical Theory and Computation 13:5683–5696.

12. Emsley, P., B. Lohkamp, W. G. Scott, and K. Cowtan, 2010. Features and development of Coot. Acta Crystallographica. Section D, Biological Crystallography 66:486–501.
